# Comparative genomics and expression analysis of polyamine oxidase gene family in *Sorghum bicolor* reveals functional specialization, gene duplication, and role in drought resilience

**DOI:** 10.1186/s12864-025-12125-4

**Published:** 2025-10-28

**Authors:** Heba T. Ebeed

**Affiliations:** 1 Botany and Microbiology Department, Faculty of Science, Damietta University, Damietta, 34517 Egypt; 2 National Biotechnology Network of Expertise (NBNE), Academy of Scientific Research and Technology (ASRT), Cairo, Egypt; 3 Council of Education and Scientific Research Policy (CESRP), Qualitative Councils Sector, ASRT, Cairo, Egypt

**Keywords:** Gene expression, Genome-wide, Polyamine oxidase, Sorghum, promoters

## Abstract

Polyamine oxidases (PAOs) are enzymes degrading the polyamine molecules and have important roles in plant growth, development and in stress tolerance. Despite their significance, their genomic organization and functional roles in *Sorghum bicolor*, a drought-tolerant staple crop, remain largely unexplored. In this study, a comprehensive comparative genomics analysis was conducted and identified six *PAO* genes in sorghum phylogenetically clustered into four clades, with sorghum exhibiting lineage-specific expansion via segmental and tandem duplications. Structural modelling identified conserved FAD-dependent oxidase cores across all SbPAO proteins and identified a novel motif (GLRLYRTSGDNSVLYDHDLEDYALYDYEGAQVPRETVLK) unique to sorghum PAOs, potentially linked to flavin-dependent oxidoreductase activity. SbPAO5 and SbPAO6 exhibited the most elaborated fold in three-dimentional modeling. SbPAO4 and SbPAO5 possess peroxisomal targeting signals (PTS1). These structural and targeting divergences collectively suggest subfunctionalization within the SbPAO family. Collinearity analysis highlighted strong syntenic conservation with rice and maize suggesting evolutionary and functional conservation of *PAOs* in grasses. Promoter sequence analysis revealed presence of several stress and hormones responsive elements, aligning with tissue- and genotype-specific expression patterns under drought. In the tolerant genotype Dorado, *SbPAO4–6* were dynamically upregulated in leaves and grains, correlating with spermidine accumulation and enhanced stress resilience. Elevated spermidine level in sensitive genotype Giza 15 suggested back conversion of spermine to spermidine by upregulation of *SbPAO5*. Co-expression analysis linked *SbPAO5* and *SbPAO6* to stress signalling and metabolic hubs implicating their roles in integrated stress adaptation. These findings establish a foundation for genomic organization and evolutionary relationships of *PAO* genes in sorghum and prioritizes *SbPAO5* and *SbPAO6* as candidates for stress-resistant breeding and enhanced production in sorghum.

**Supplementary Information:**

The online version contains supplementary material available at 10.1186/s12864-025-12125-4.

## Introduction

The polyamines (PAs) are naturally occurring molecules characterized by two or more amino groups. The most spread PAs are Putrescine (Put), Spermidine (Spd), and Spermine (Spm), which all differ from each other concerning the number of amino groups. Senescence [1], flowering [2], fruit ripening [3], and stress responses [4–6] were all shown to be significantly affected by PAs. Enzymes called polyamine oxidases (PAOs) have important roles both in plant growth and development and in stress tolerance. The oxidation reaction that PAOs catalyze to generate products such as H_2_O_2_ may activate a signaling cascade that modulates the activities of downstream responsive proteins favorably [7–9]. Inevitably, PAO activity will impact the titers of various PAs, which in turn will impact plant responses to stressful conditions. As shown by Wu and colleagues [10], this ultimately impacts plant defense responses. Besides, the PAOs degrading PAs exhibit functional diversity. The first group of PAOs has roles in the terminal catabolism of PAs, undertaking the oxidation and breakdown of Spd and Spm to produce H_2_O_2_, 1,3-diaminopropane and 4-aminobutanal in the case of Spd or N-(3-aminopropyl)−4-aminobutanal in the case of Spm catabolism [11, 12]. The second class of PAOs, on the other hand, mediates the back-conversion reactions of PAs, that is, Spm to Spd and Spd to Put. It needs to be mentioned here that such a reverse process of PA biosynthesis generates 3-aminopropanal and H_2_O_2_ as by-products as well. As reported by Wang et al. [13], some PAO proteins are peroxisomal as for three members of Arabidopsis PAO: AtPAO2, AtPAO3, and AtPAO4 whereas, AtPAO5 and AtPAO1 are found in the cytoplasm [14]. Likewise, TaPAO1 is a cytoplasmic protein and OsPAO3-5 is reportedly peroxisomal [15]. A C-terminal peroxisomal targeting sequence I, PTS1, is present in these proteins [16]. The PTS1 consists of a tripeptide with the consensus sequence (S/A/C)(K/R/H)(L/M) [16]. OsPAO6, OsPAO7, and ZmPAO1 are also localized in the apoplast [17–19].

Genome-wide analyses have identified *PAO* gene families in various plant species, including maize [20], pear [21], grape [22], potato [23], and citrus [24]. These studies reveal based on their structural characteristics and phylogenetic relationships [20]. Structurally, plant PAOs are FAD-dependent oxidases characterized by a conserved fold featuring a FAD-binding domain (Rossmann fold) and a substrate-binding domain [25, 26]. Key catalytic residues (e.g., a conserved active-site Tyr/Glu pair involved in substrate orientation and a Lys stabilizing FAD) are conserved across species [27]. Structural analyses have revealed determinants of substrate specificity (terminal vs. back-conversion) and subcellular adaptation, such as variations in surface loops potentially influencing protein-protein interactions or organellar targeting [15, 28].

Sorghum serves as both staple food and animal feed and has capacity to thrive in arid conditions. It ranks as the fifth most produced cereal grain in the world. The exceptional capacity of sorghum to survive hot and arid conditions makes it, within the grass family, an exceptionally good model for drought resistance studies. Consequently, current sorghum breeding efforts are focused on enhancing its adaptation to climate change, primarily targeting biotic and abiotic stresses [29]. Advances in genome sequencing technologies, databases, and bioinformatic and comparative genomics tools have greatly facilitated the identification and understanding of the molecular mechanisms underlying stress tolerance in plants [30–32]. As *PAO* genes are very important in stress response and haven’t been studied in sorghum earlier and particularly under drought stress. Recently, using sequence-based identification approach we identified *PAO* in sorghum without deep structural and functional analyses [33]. The present study incorporating comparative genomic and phylogenetic analyses, structural modeling of SbPAO proteins, evolution and collinearity of the gene family and detailed gene expression profiling under drought stress. Importantly, further validated the functional roles of key *PAO* genes through polyamine quantification, and analysis of agronomic performance under drought conditions. Together, these integrative approaches provide new insights into the biological significance and potential application of *SbPAO* genes; particularly *SbPAO5* and *SbPAO6* in sorghum drought resilience.

## Materials and methods

### Sequence retrieval and protein sequence analyses

Protein sequences of *SbPAO* identified genes in Ebeed et al. [33] were retrieved from *S. bicolor* genome v3.1.1 on phytozome database (https://phytozome-next.jgi.doe.gov/). To identify conserved motifs, each homolog protein sequence was scanned for motifs using an online MEME tool-package known as Multiple Expectation Maximization for Motif Elicitation available at http://meme.sdsc.edu/meme/meme.html. The parameters were set to the following: maximum 10 motifs with width between 6 and 50 amino acids. Protein-protein interaction (PPI) map of the predicted PAO proteins and with other proteins were generated from the STRING database [34].

To further understand the protein structure of the SbPAO family, 3D models for all SbPAOs were predicted based on their protein sequences, and the 3D structures of the proteins were built using homologous protein modelling on the Swiss-Model website (https://swissmodel.expasy.org). The subcellular localization of each wheat protein was predicted using the Plant-mPloc tool developed by Chou and Shen [35]. The PredPlantPTS1 method developed by Reumann et al. [36] was used to search for peroxisomal targeting signal 1. Membrane-bound proteins in the results were further verified by TMHMM-2.0 described by Krogh et al. [37]. With the help of Predotar and the same features as above by Small et al. [38] and TPpred 3.0 by Savojardo et al. [39], the putative N-terminal targeting sequences were also addressed. ApoplastP methodology as developed by Sperschneider et al. [40] was used to assess possible apoplastic localization.

### Comparative and evolution analysis of PAO in sorghum

Phylogenetic analyses were performed and the Bayesian inference methods. Using Clustalw in MEGA X [41], multisequence alignment was performed with the 6 protein sequences. Then, geneDoc software [42] was used to calculate and analyze the conserved sequences of the SbPAO family. The phylogenetic tree of the PAO protein family in sorghum, maize, rice, and Arabidopsis was constructed using the amino acid sequences with the yeast as outgroup by the adjacency joining algorithm in MEGA X software. The bootstrap test was carried out with 1,000 iterations (replications). The obtained evolutionary tree was further modified using the iTOL (https://itol.embl.de/).

To trace the sequence similarity patterns, TBtools software was used for synteny analysis and visualization of circos plot for the *SbPAO* genes. Multiple Collinearity Scan toolkit (MCScanX) [43] with default parameters was used for analyzing the gene duplication events. To trace the homology of the *PAO* gene family between sorghum and other species, the Dual Systeny Plotter software (https://github.com/CJ-Chen/TBtools) was used for mapping intergenomic collinearity analysis.

### Chromosome mapping and analysis of promoter of PAO genes

All *PAO* genes were mapped to their chromosomal positions in four chromosomes using the chromosome mapping tool (http://mg2c.iask.in/mg2c_v2.1). The locus position of the *PAO* gene and chromosome length was extracted according to annotation information about the *S. bicolor* genome, V3.1.1.

Promoter sequences for all *PAO* genes were obtained from the JGI database, 2 kb upstream of the 5’UTR. The promoter regions were screened for *Cis*-acting Regulatory Elements (CREs) prediction through PlantCare [44]. The identified CREs were then compared across the different *PAO* genes and their importance discussed in respect to the available literature.

### In Silico RNA sequence expression and co-expression analyses of PAO genes

RNA-Seq data were obtained from SRA (Sequence Read Archive) available at the NCBI database and were used solely for comparative purposes. To investigate the expression patterns of *SbPAO* genes in different tissues. RNA sequencing data (PRJDB1973; DRX027768:DRX027773) of seed and spikelet tissues of *S. bicolor* (Btx623) (Makita et al., 2015) were analyzed to study the gene expression of the tissues at seed forming stage. RNA sequencing dataset (ERP024508) [45] were downloaded to analyze the expression level of *SbPAO* genes in leaf, root, shoot and whole seedling at 14 days after germination. Gene expression values were counted by Transcript Per Million (TPM) as average for each set of technical replicates, then quantile normalized within each set of biological replicates using limma. Finally, they are averaged for three biological replicates.

To study the expression patterns of *SbPAO* genes against abiotic stresses, the array expression profile data (GSE48205) for response to heat and drought stress both individually, and in combination were explored [46]. The gene expression values were calculated by Fold Change (FC). Differential expression analyses were performed by comparing the expression of a gene at each treatment to control.

Co-expression analysis was done for *SbPAO* genes using sorghum functional genomics database (SorghumFDB; http://structuralbiology.cau.edu.cn/sorghum/index.html) [47] that contains sorghum experiments with multidimensional biological relationships of co-expression data, protein–protein interactions and miRNA-target pairs from all tissues.

### Plant material and abiotic stress treatments

Two cultivars of *S. bicolor* (Dorado and Giza-15) with contrasting responses to drought stress have been used. Seeds were sown in rows with spacing of 20 cm. The agriculture practices followed used the recommended local sorghum agriculture, except for irrigation treatments throughout the growing seasons. Control plants irrigated every 10–15 days. Drought stress was imposed by withholding irrigation for three weeks just before flowering (60 days after sowing). Leaves and grains tissues were collected for gene expression analysis. The middle part of the flag leaves was collected from randomly sampled treated and untreated control plants 90 days after sowing while the grains were harvested at 100 days after sowing. Grain yield (wt of 1000 grains) was recorded from nine randomly selected plants from the inner middle rows of the plot. Soil samples have been collected at harvest days to assure difference in soil moisture content between control and drought-exposed plants. Soil moisture content wasdetermined by subtracting the dry weight of soil sample from fresh weight and dividing by the dry weigh. Harvested materials were directly immersed in liquid nitrogen and transferred for storage to a −80˚C deep freezer for subsequent use. Three biological replicates and three technical replicates each were employed in qRT-PCR studies.

### Measurement of free polyamines

The plant sample (leaves and grains) weighing 0.5 g was taken and pulverized in ten volumes of cold perchloric acid 5% (v/v) for 1 h at ice temperature. After this period, centrifugation was performed at 15,000×g for 30 min at 4 °C, and the supernatants were collected. To 1 ml of the extract and 1 ml of 2 N NaOH, add 10 µl of benzoyl chloride, then vortex for 10 s. Add saturated NaCl and diethyl ether, both 2 ml each, mix vigorously, and centrifuge at 3000×g for 10 min at room temp. PAs were analyzed by HPLC at 254 nm, a flow rate of 1 ml/min. For separation and column washing, 42% acetonitrile was used.

### RNA isolation and quantitative real-time PCR analysis

qRT-PCR analysis was performed on leaves and grains under drought/salinity stress. Leaves were prioritized as primary stress-responsive organs where PAO-mediated H₂O₂ signaling occurs, while grains represent key yield components. Total RNA was isolated from the samples of flag leaves and grains that were exposed to stress and control (no stress) samples using Triazole reagent (Bioline), according to the instructions. The RNA samples were cleaned of genomic DNA contamination using DNase I (Thermo Scientific). Using the Sensifast 1 st cDNA synthesis kit, Bioline, two micrograms of RNA sample served as the template for the first strand cDNA synthesis. For the determination of relative gene expression levels of *SbPAOs*, gene specific primers (Table S1) with SensiFast SYBR Lo-Rox (Bioline) 2X Master Mix were used. Specific primers were designed using Primer3 and checked for specificity for each template using Primer Blast. For that, the thermal cycling conditions were 95˚C for 5 min, followed by 40 cycles of 95˚C for 30 s, 57˚C for 30 s, and 72˚C f or 30 s in the STRATAGENE MxPro-3000P real-time PCR system. Expression of *SbPAO* genes was normalized by *GAPDH* reference genes in both controls and treatments. *GAPDH* (Sobic.005G159000) evaluated as candidate reference genes using transcriptome screening in mRNA-seq: GSE54705; https://www.ncbi.nlm.nih.gov/geo/query/acc.cgiacc=GSE54705 and mRNA-seq: GSE50464; https://www.ncbi.nlm.nih.gov/geo/query/acc.cgiacc=GSE50464 (Figure S1) and consistency of its quantification cycle (Cq) values across all samples in qRT-PCR. In accordance with MIQE guidelines [48], each sample were done in three biological and three technical duplicates and the PCR amplification efficiency was determined for each primer combination automatically calculated by the MxPro – Mx3000P software using the input information of standard concentrations and dilutions used into the program. The standard curve was 5 serial dilutions of a mixture of all sample cDNAs. The PCR efficiencies ranged from 90 to 110%. The Melting curve examination of the amplicons verified the specificity of the PCR reaction. The relative amounts of each transcript were determined using the comparative ΔΔCT method [49].

### Statistical analyses

To find the significant changes between samples at *P* < 0.05, two-way ANOVA was used. To compare significant effects, least significant difference (LSD) test was employed with a *P* < 0.05.

## Results

### Protein sequence analyses

MEME online software was used to predict the conserved motifs in the six SbPAO protein sequences and as a result, 10 motifs were identified. The width of motifs ranges from 20 (motif 10) to 50 AAs (motifs 2 and 4) (Table S2). Motif sequences and description were also shown in Table S2. Six motifs were identified to be Flavin containing amine oxidoreductase (motifs numbers 1, 2, 3, 5, 7, and 8) while four motifs were not found in Pfam or any motif identifier database (motifs no. 4, 6, 9, and 10). As shown in motifs distribution pattern in (Figure S2), motifs no. 3, 5, 6, and 7 were distributed to all the SbPAO proteins and are the most frequent motifs. Motifs no. 1, 2, 8, and 10 present in all sequences except SbPAO6. Motifs no. 4 and 9 present only in SbPAO3-5. The predicted motif sequence no. 6 could be novel motif for flavine containing amine oxidoreductase as it is conserved in all identified SbPAO proteins.

**Fig. 1:**
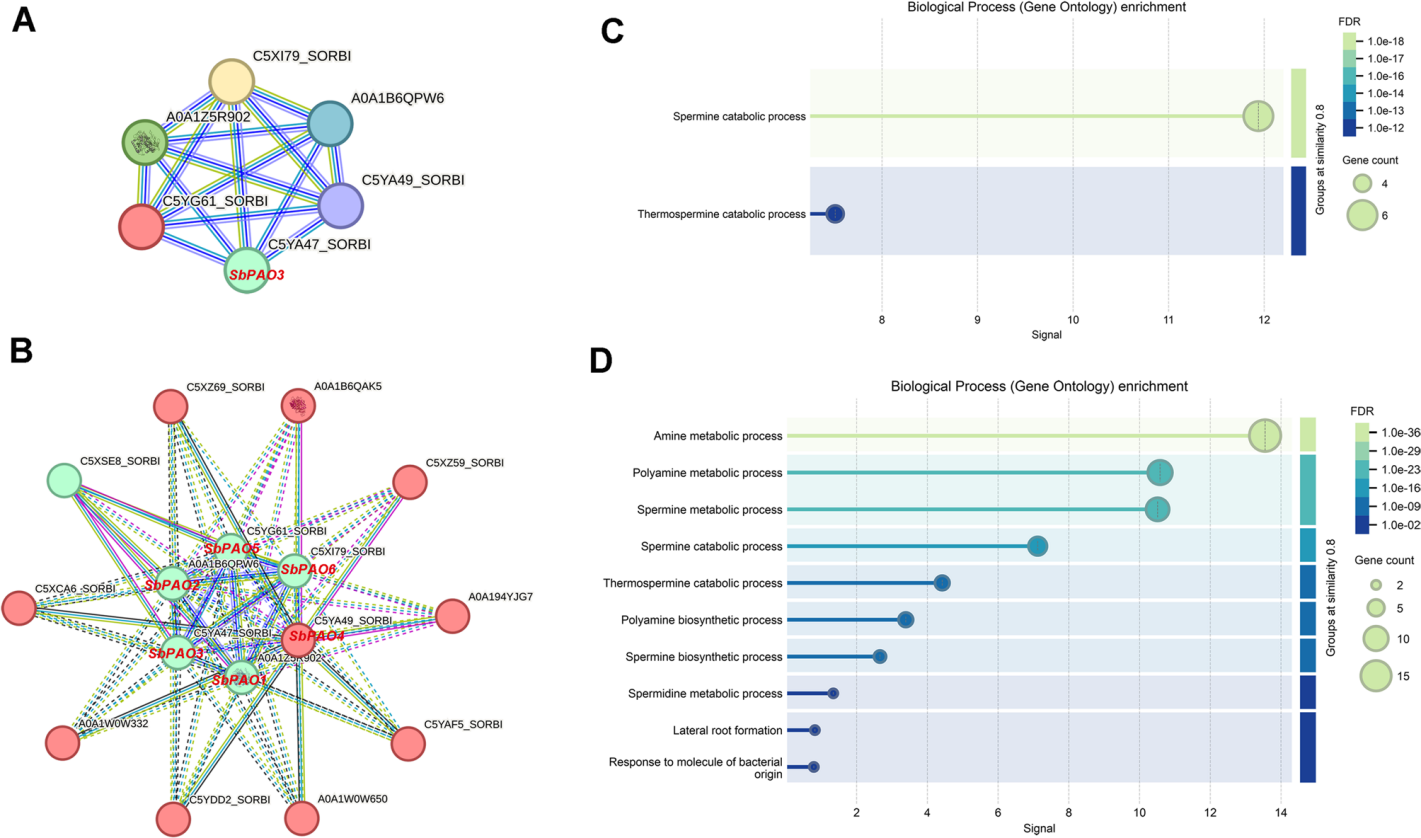
Protein-Protein Interaction (PPI) Network and Gene Enrichment Analysis of PAO Proteins in *Sorghum bicolor. *(**A**) STRING analysis of the predicted PAO proteins. A PPI network at high confidence showing interactions among members of the PAO family is presented. Nodes represent PAO proteins, and edges represent predicted interactions that are supported by experimental data, co-expression, and conserved functional associations. (**B**) Extended PPI network; the interactions between PAO proteins with other predicted partners participating in related metabolic pathways are shown. (**C**) GO enrichment analysis of biological processes associated with the predicted PAO proteins. Among them, spermine catabolic process and thermospermine catabolic process are significantly over-represented. (**D**) GO enrichment analysis of biological processes for the expanded PPI network. Over-represented processes include polyamine metabolic process, polyamine biosynthetic process, and lateral root formation. In panels (**C**) and (**D**), the circle size corresponds to gene number of each process, while color gradient corresponds to the statistical significance by FDR value

The predicted PAO proteins in sorghum and their protein-protein interaction network along with their functional enrichment analysis are represented in (Fig. 1 and Table S3). The upper-left network (Fig. 1A) represents the interactions within the six predicted PAO proteins with high-confidence scores. The lower left network in Fig. 1B extends the interaction map to additional predicted partners. Legend figure indicating nodes colour and details of predicted functional partners in Supplementary Figure S3. Here, nodes represent individual PAO proteins, while edges denote interactions between them as predicted by STRING analysis. The upper right panel (Fig. 1C) represents GO biological process enrichment for the core PAO proteins. It marks processes such as spermine catabolic process and thermospermine catabolic process, which substantiate their crucial roles in polyamine metabolism. It reveals associations with other proteins that participate in the extended metabolic processes, for example, amine and polyamine metabolism. The lower right panel (Fig. 1D) extends the enrichment analysis to associated proteins of the extended PPI network. The enriched biological processes include amine metabolic process, polyamine biosynthetic and metabolic processes, and responses to environmental stimuli, such as bacterial molecules.

**Fig. 2:**
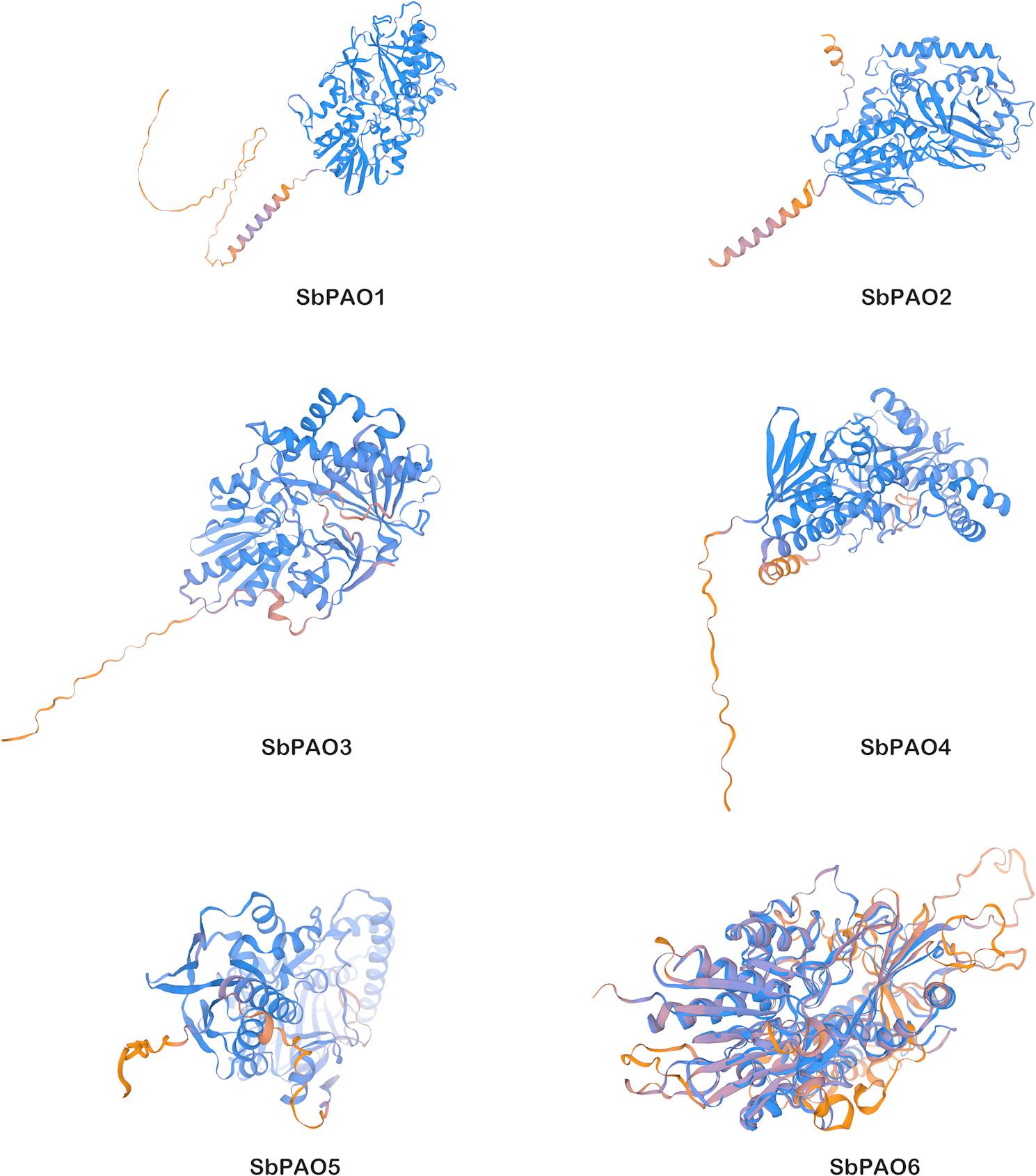
Predicted 3D Structures of Polyamine Oxidase (PAO) Proteins in *S*. *bicolor *(SbPAO1–SbPAO6). Colors denote secondary structure: helix (blue) and β-sheets (red). Models were generated using Swiss-Model (Expasy)

Predicted 3D structures of PAO proteins in sorghum are shown in Fig. 2. Each model exhibits unique structural characteristics and potential functional zones. 3D structures of SbPAO1 and SbPAO2 show a compact globular core of tightly packed α-helices and β-sheets, characteristic of FAD-dependent oxidases. Longer loops and coiled regions at the termini hint at flexible domains that may interact with the substrates or cofactors. Like SbPAO1 and SbPAO2, SbPAO3 and SbPAO4 models also exhibit a conserved catalytic core but with protruding helices. On the other hand, SbPAO5 structure has a highly intricate fold with visible interactions between helices and sheets, suggesting potential stability. Most interestingly, SbPAO6 model presents the most complex fold among the six proteins with extensive secondary structure elements and interconnected regions.

Knowledge of subcellular localization of sorghum PAO proteins was determined in this study based on various prediction tools. The main tool that was employed was Plant mPLoc, the prediction with respect to the subcellular localizations indicated in Table 1 revealed that all sorghum PAO proteins localized in the chloroplast. To further validate the subcellular localizations and to support the above predictions, Predator and TargetP were used. Surprisingly, the predictor tools showed contrasting subcellular localization for some PAO proteins. Specifically, SbPAO2 was predicted to localize to the endoplasmic reticulum while SbPAO6 was predicted to localize to the mitochondria. By prediction assessments, it was shown that SbPAO4 and SbPAO5 were localized to the peroxisome because they contain peroxisomal targeting signal 1 (PTS1) and none of PAOs were apoplastic and PAO3-6 were predicted to be transmembranous proteins.

**Table 1:** The prediction of subcellular localization and peroxisomal targeting signals (PTS) for sorghum PAO proteins

Sorghum proteins	Plant-mPLoc Localization	Predotar, TargetP	Trans-membrane	Apoplast	PTS1
SbPAO1	Chloroplast	none	-	Non	-
SbPAO2	Chloroplast	ER	-	Non	-
SbPAO3	Chloroplast	none	√	Non	-
SbPAO4	Chloroplast	none	√	Non	PTS1
SbPAO5	Chloroplast	none	√	Non	PTS1
SbPAO6	Chloroplast	Mito	√	Non	-

### Genome organization and evolutionary analysis of SbPAO genes

The multiple sequence alignment (MSA) of *S. bicolor* PAO proteins reveals conserved regions and sequence similarities between six different PAO members (Figure S3). Highly conserved residues are shaded in black, showing their importance in the functioning of PAO. Moderately conserved residues are shaded in gray and red residues for PTS1 in SbPAO4 and SbPAO5.

**Fig. 3:**
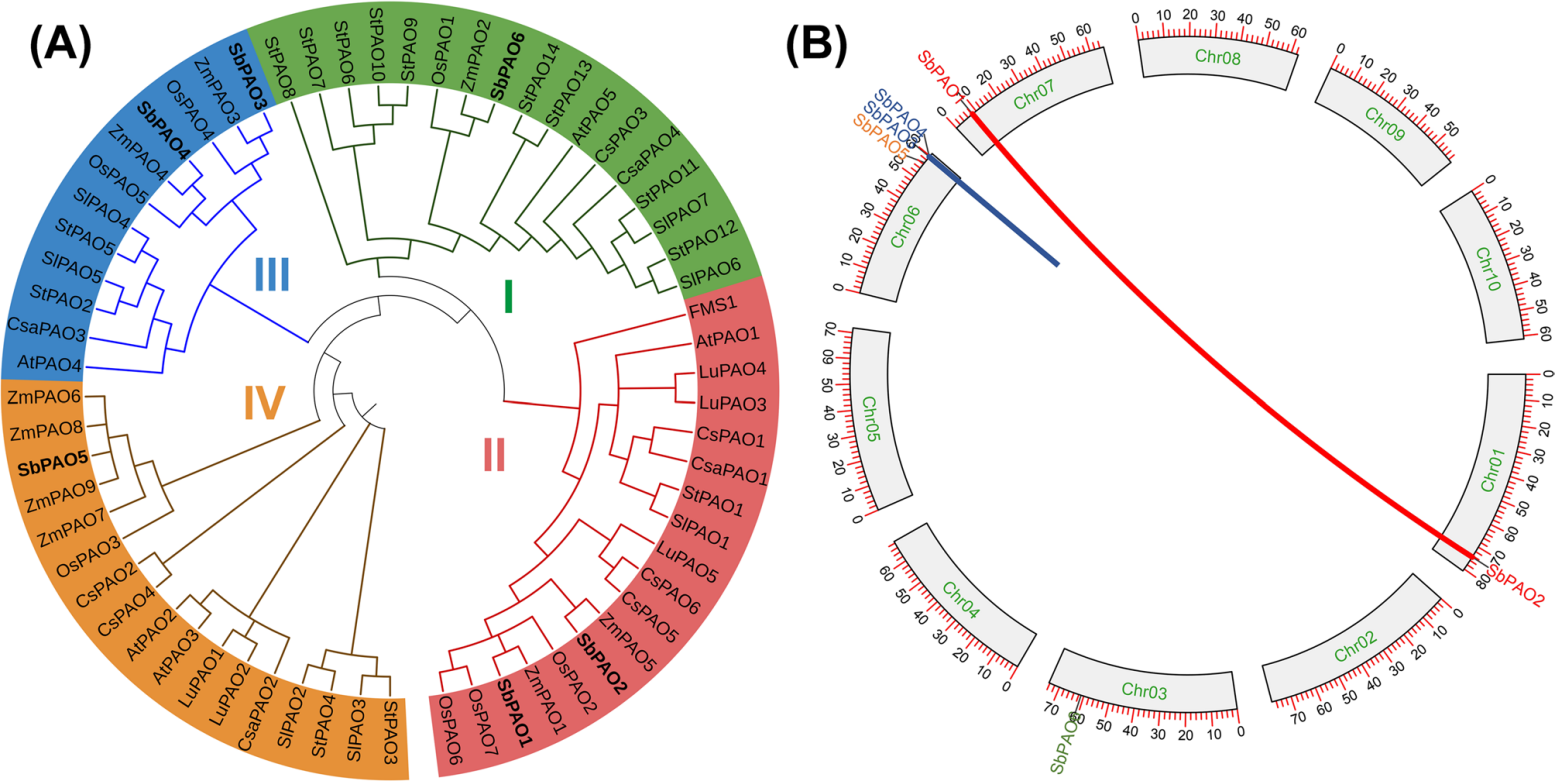
**A** Phylogenetic analysis of PAO proteins in sorghum, maize, rice and *Arabidopsis*. The tree was constructed using the neighbor-joining method using MEGA X software. Different clades are marked with distinct colors, showing evolutionary relationships and potential functional diversification among PAO proteins across these species. The phylogenetic tree (1000 replicates) was constructed by NJ method and bootstrapping analysis. *At* Arabidopsis thaliana, *Sb* Sorghum bicolor, *Zm* Zea mays, *Os* Oryza sativa, *Sl* Solanum lycopersicum, *Cs* Citrus sinensis, *Lu* Linum usitatissimum, *St* Solanum tuberosum, *Csa* Cucumis sativus, *FMS1* PAO of *Saccharomyces cerevisiae* used as outgroup. Gene IDs are represented in Supplementary Table S4. (**B**) Circos plot illustrating the chromosomal distribution and syntenic relationships of *PAO* genes in *S. bicolor*. The outermost circle represents the sorghum chromosomes, Chr1- Chr10. The positions of *PAO* genes are marked with labels and scattered over chromosomes 1, 6, and 10. Segmental duplication between *SbPAO1* on Chr7 and *SbPAO2* on Chr1 is represented by red connecting lines. *SbPAO3*, *SbPAO4*, and *SbPAO5* tandemly clustered together on chromosome 6 indicates tandem duplication events represented by the blue line

The phylogenetic tree represents the evolutionary relationships among the PAO proteins from sorghum, SbPAOs; Arabidopsis, AtPAOs; maize, ZmPAOs; and rice, OsPAOs. This tree is divided into four major clades with one color for each (Fig. 3A). Sorghum proteins were distributed in the four clades as maize and rice proteins on contrast to Arabidopsis that classified into three clades I, II and IV. SbPAO6 belongs to group I, SbPAO1 and SbPAO2 to group II, SbPAO3 and SbPAO4 to group III and SbPAO5 to group IV.

The circos plot in Figure 3B illustrates the chromosomal distribution and syntenic relationships of *PAO* genes in the sorghum genome. *PAO* genes are distributed on four chromosomes. A segmental duplication event is suggested between *SbPAO1* (Chr7) and *SbPAO2* (Chr1), as indicated by the red connecting line. The clustering of the three *PAO* genes, *SbPAO3*, *SbPAO4*, and *SbPAO5*, on chromosome 6 suggests tandem duplication events, as is common for expansions of gene families.

**Fig. 4:**
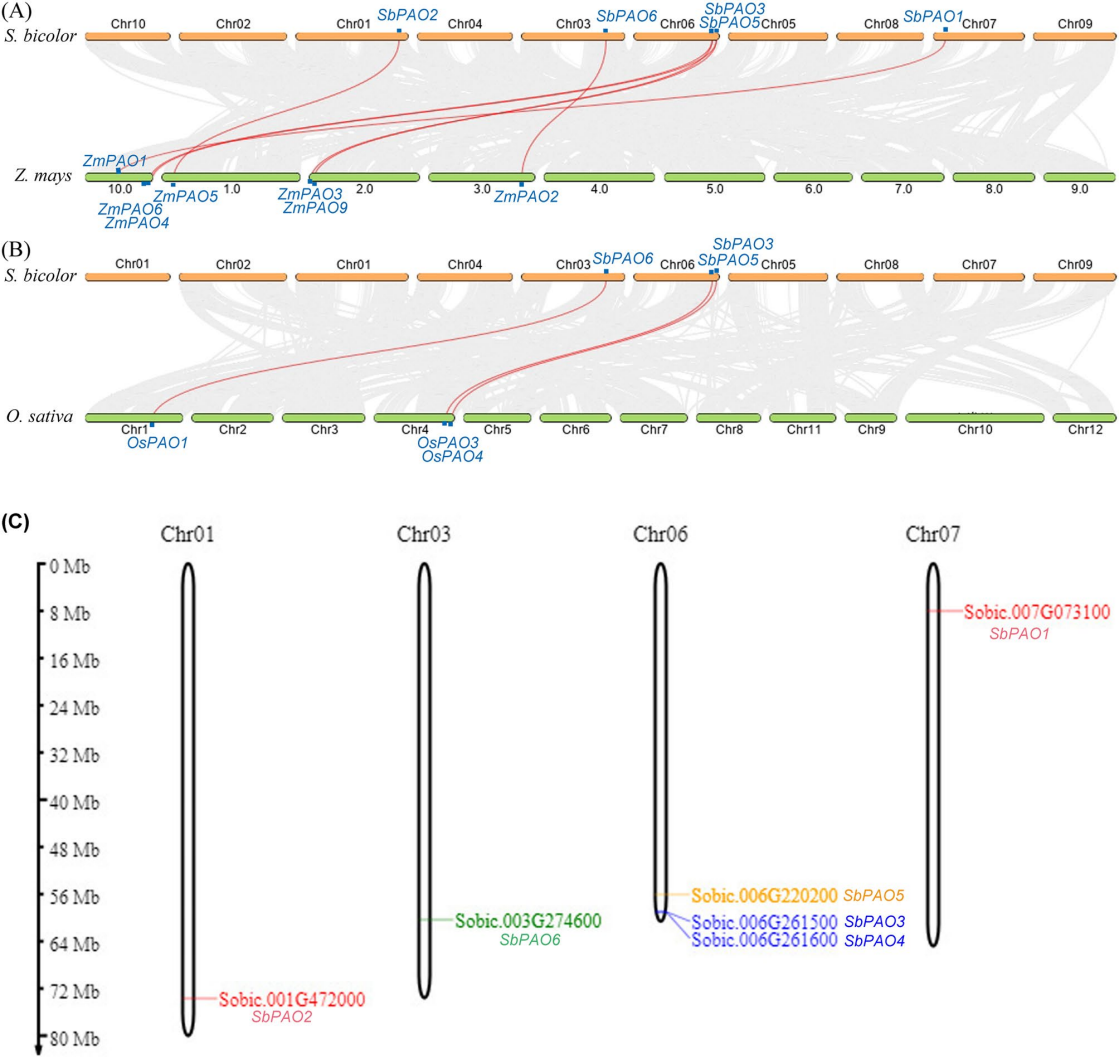
Synteny and genomic positions of *PAO* genes. (**A**) Syntenic relationships between *S. bicolor* chromosomes (orange bars, Chr01–Chr10), *Zea mays* chromosomes (green bars, Chr01–Chr10) (**A**) and *Oryza sativa* (**B**) are shown with connecting arcs. Grey arcs indicate conserved syntenic blocks across the genomes and red arcs highlight positions of conserved *PAO* genes between sorghum and maize. (**C**) the chromosomal locations of *PAO* (polyamine oxidase) genes within the sorghum genome. *PAO* genes are mapped to distinct regions on chromosomes Chr01, Chr03, Chr06, and Chr07. The scale on the left is in megabases

To understand the evolutionary conservation and genomic organization of the *PAO* genes, synteny analysis of the *PAO* genes were employed with sorghum and other species (Fig. 4A&B). Syntenic relationship between chromosomes of sorghum (orange bars) and maize or rice (green bars) is represented in the Fig. 4A&B. The arcs in the figure connect regions of homologous genes shared between the two species and show conserved syntenic blocks. Red arcs represent the *PAO* genes. Table 2 highlights collinearity of *PAO* genes in sorghum with other species. The results showed that five *PAO* genes have seven collinearity relationship with maize genome (Table 2) and interestingly, each of *SbPAO3* and *SbPAO5* has two collinear relationships with *Z. maize* genome and no relationship between *SbPAO4* and maize genes, while only three collinear gene pairs were revealed by the analysis between sorghum and rice.

**Table 2:** The Colinear *PAO* gene pairs in sorghum and maize or rice

Collinearity between Sorghum bicolor and Zea mays			
Chromosome	S. bicolor Gene ID	Chromosome	Z. maize Gene ID
Chr07	Sobic.007G073100_*SbPAO1*	10	Zm00001d024281_ *ZmPAO1*
Chr01	Sobic.001G472000_ *SbPAO2*	1	Zm00001d028172_ *ZmPAO5*
Chr03	Sobic.003G274600_ *SbPAO6*	3	Zm00001d043681_ *ZmPAO2*
Chr06	Sobic.006G220200_ *SbPAO5*	10	Zm00001d026334_ *ZmPAO6*
Chr06	Sobic.006G261500_ *SbPAO3*	10	Zm00001d026586_ *ZmPAO4*
Chr06	Sobic.006G220200_ *SbPAO5*	2	Zm00001d002266_ *ZmPAO9*
Chr06	Sobic.006G261500_ *SbPAO3*	2	Zm00001d001883_ *ZmPAO3*

Collinearity between ***Sorghum bicolor*** and ***Oriza sativa***			
Chromosome	***S. bicolor*** Gene ID	Chromosome	***O. sativa*** Gene ID
Chr03	Sobic.003G274600_ *SbPAO6*	Chr1	Os01g51320_*OsPAO1*
Chr06	Sobic.006G220200_ *SbPAO5*	Chr4	Os04g53190_*OsPAO3*
Chr06	Sobic.006G261500_ *SbPAO3*	Chr4	Os04g57550_ *OsPAO4*

### Chromosome location and promoter analysis of SbPAO genes

To understand the genomic distribution and organization of the *PAO* genes in the sorghum genome, the genes were mapped to the chromosome and presented in Fig. 4C. As seen in the following figure, there are *PAO* genes on four chromosomes, but the distribution is fragmented to Chr01, Chr03, Chr06, and Chr07.

**Fig. 5:**
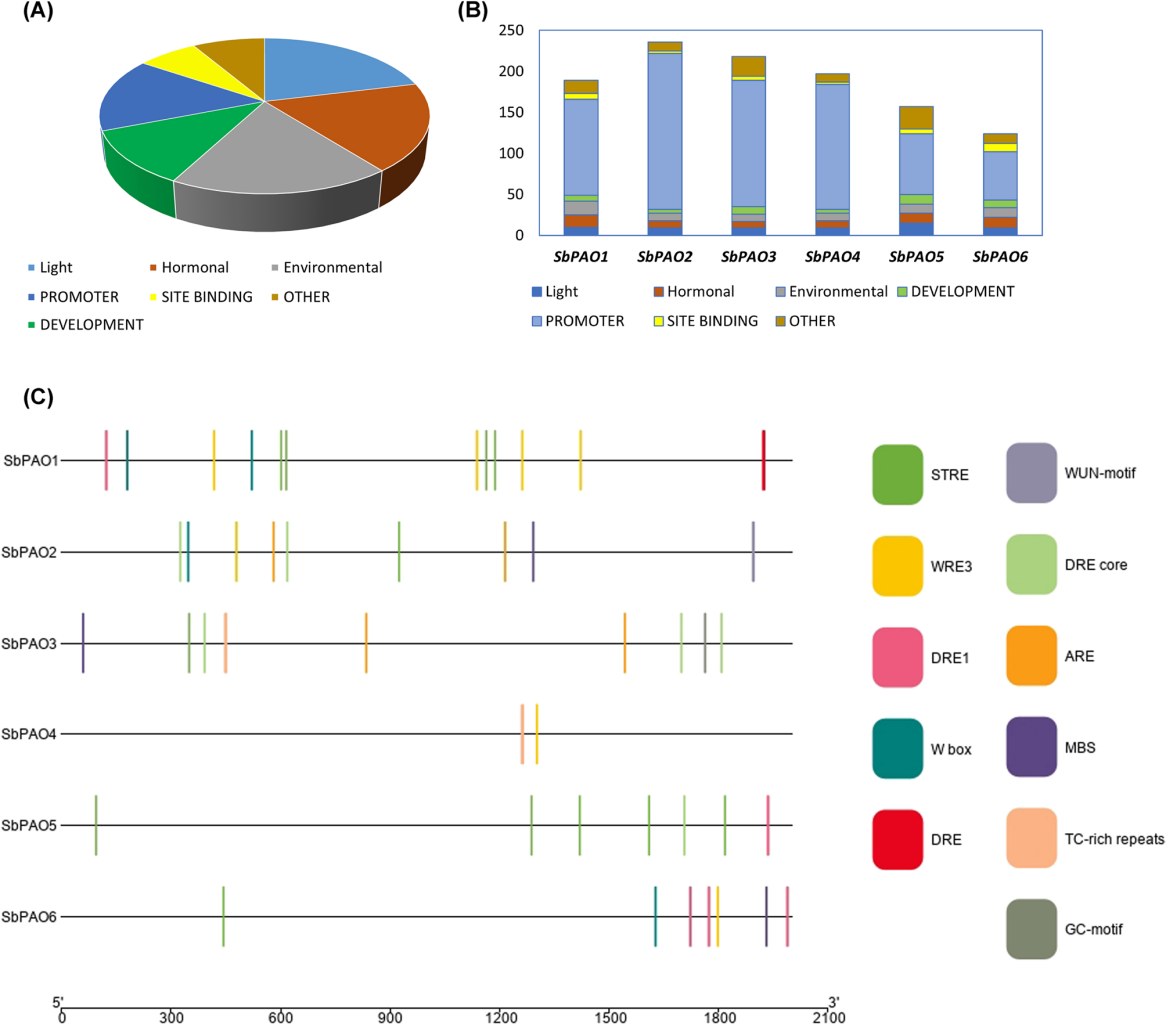
*Cis-*regulatory elements in the sorghum *PAO* gene promoter region. (**A**) pie chart representing the total of the 71 CREs found in sorghum *PAO* promoters categorized into seven groups. (**B**) Location of the discovered stress responsive elements in the wheat 2000 bp promoter area. (**C**) Distribution of each CREs category in each sorghum promoter; each CREs category’s number of elements is shown in the histogram with a different color

**Fig. 6:**
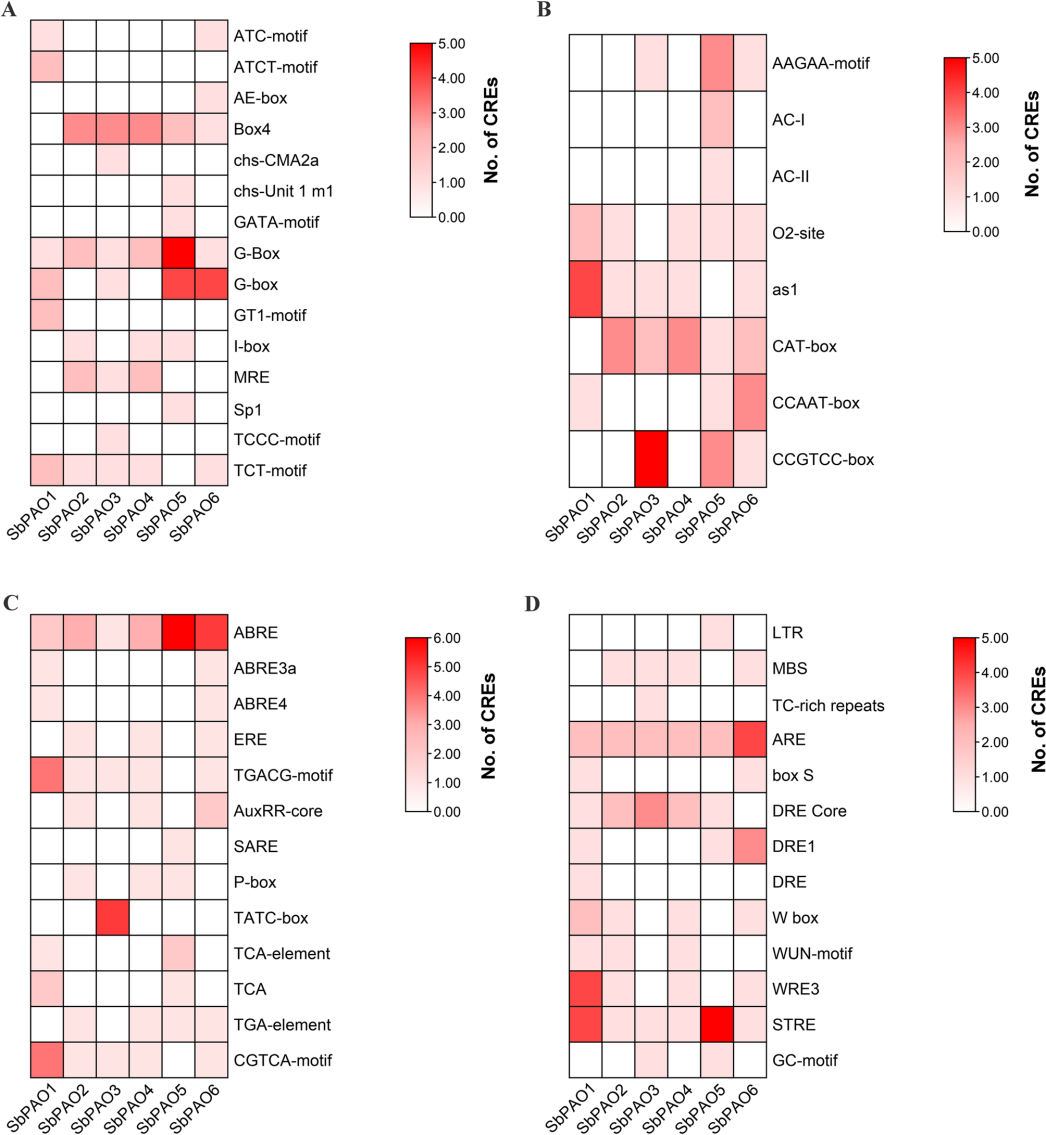
Heatmap showing the frequency of *Cis-*regulatory elements (CREs) in the sorghum *PAO* gene promoter region. (**A**) Frequency of light responsive elements of the *SbPAO* promoters. (**B**) Frequency of development related elements of the *SbPAO* promoters. (**C**) Frequency of hormone related elements of the *SbPAO* promoters. (**D**) Frequency of stress responsive elements of the *SbPAO* promoters

CREs play important roles in the regulation of gene expression. CREs in the promoter sequences for each gene were identified using the PlantCARE database. A total of 71 unique CREs were found (Supplementary Table S5). These CREs were grouped into the seven classes of promoter-related, site-binding related, light-responsive, hormone-responsive, environmental-responsive, developmental, and other elements with unknown roles (Fig. 5A). All classes of CREs were present within the promoters of the sorghum *PAO* genes, although at different frequencies (Fig. 5B). The locations of the environment responsive elements in the 2000 bp promoter sequences that come before each gene’s ATG position are shown in Fig. 5C. The number and cumulative count of predicted CREs in the promoters of sorghum *PAO* genes are summarized in Table S6 and Table S7. Figure 6 represents heatmaps of frequency of each group of CREs within 2000 bp promoter length. Interestingly, environment responsive elements are found in all promoters, but hormone- and environment-responsive elements were more frequent in promoters of *SbPAO1*, *SbPAO5*, and *SbPAO6* and development responsive elements were more frequent in *SbPAO5*, and *SbPAO6* (Table S6 and S7). G-Box, ABRE, STRE, CAAT-box, TATA-box, MYB, and MYC were noticed in all promoters (Supplementary Table S5). These are two stress-responsive elements (STRE, ARE), four promoter-related elements (CAAT-box, TATA-box, MYB, MYC), one light responses element (G-Box), and one hormone responsive element (ABRE). Furthermore, eight elements were the most common (> 80%) in all promoters. These elements are Box4 and TCT-motif (involved in light responsiveness), TGACG-motif and CGTCA-motif (involved in the methyl jasmonate (MeJA)-responsiveness), DRE Core, as1 element which is involved in tissue-specific expression, Myb and Myb-binding site (Supplementary Table S5).

### Transcriptome data analysis for SbPAO genes

**Fig. 7:**
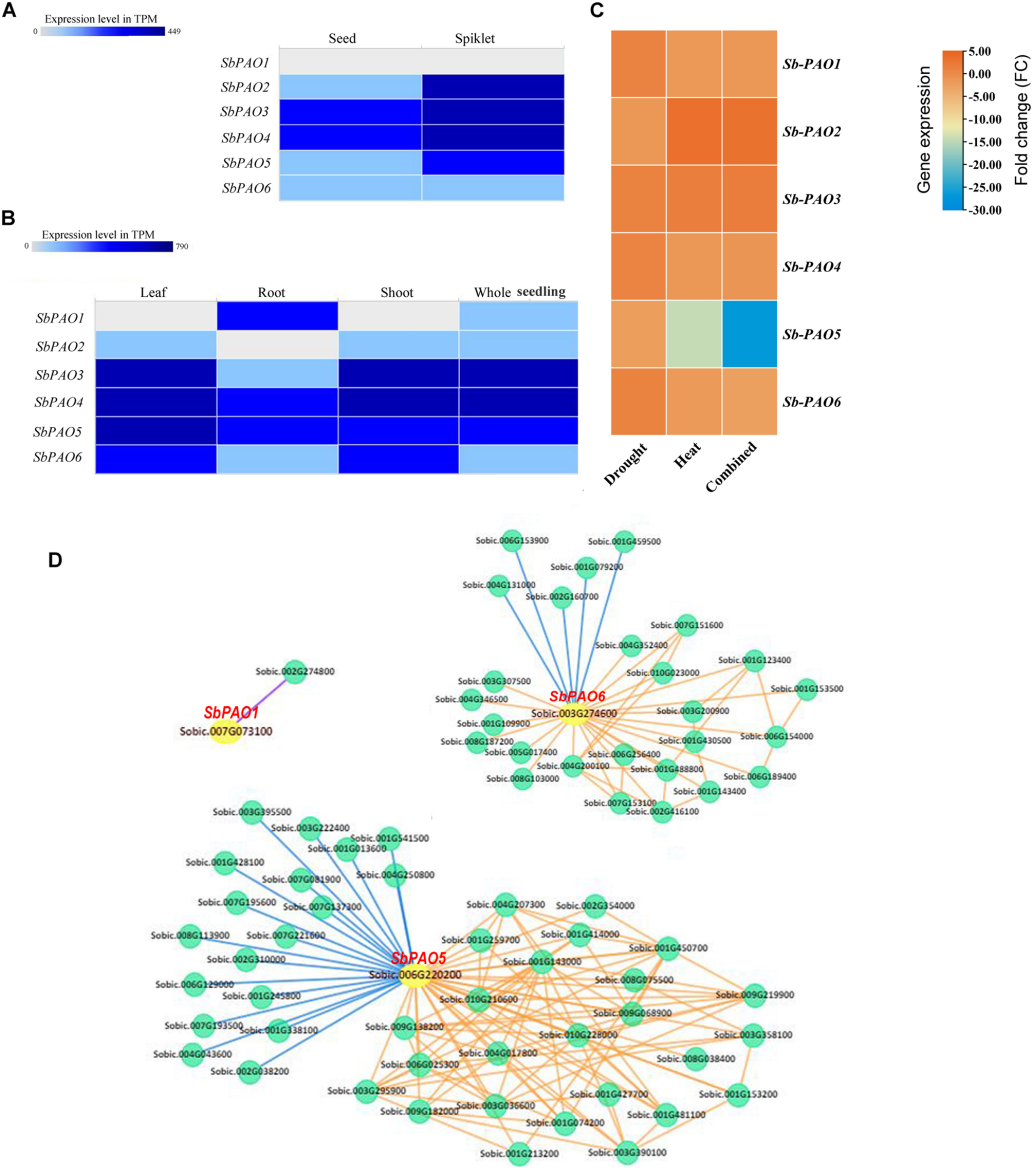
Expression profiles of *SbPAO* genes in sorghum tissues. Heat maps of *SbPAO* gene expression in seeds and spikelets (**A**) and leaves, roots, shoots, and whole organisms (**B**). Expression values are in TPM. The darker shade of blue represents a higher expression. (**C**) Heatmap of gene expression levels (fold change) of *PAO* genes in sorghum under drought, heat, and combined drought and heat stress conditions. Upregulation in orange; strong downregulation in blue. Gene names (*SbPAO1* to *SbPAO6*) on the y-axis; stress conditions on the x-axis. (**D**) Co-expression networks of *SbPAO* genes in sorghum. *SbPAO* genes are shown in red and accession numbers in yellow circles (Sobic.006G220200, Sobic.003G274600, and Sobic.007G073100). Orange and blue lines indicate positive and negative co-expressional relationship, respectively. Purple line indicates PPI

For further understanding of the molecular function of *PAO* in sorghum and in response to stressed conditions, expression profiles at the level of *SbPAO* genes were analyzed in transcriptome data sets, which are represented in Fig. 7. Figure 7 shows levels of *SbPAO* gene expressions in different sorghum tissues; seed and spikelet (Fig. 7A), leaves, roots, shoot, and whole seedling (Fig. 7B). Heatmap of gene expression level in TPM. The darker shades of blue depict higher levels of expression: *SbPAO3* and *SbPAO4* are highly expressed in seed and spikelet reproductive tissues; *SbPAO1* is strongly expressed in roots and not in shoots and leaves; *SbPAO3*, *SbPAO4*, and *SbPAO5* showed marked expression in shoots and leaves; *SbPAO6* showed a relatively high level of expression in roots and shoots.

Expression levels of *PAO* genes in sorghum under different stress conditions, namely, drought, heat, and combined drought and heat, are shown in Fig. 7C. The heat map is colored by changes in the gene expression levels; the color orange represents the up-regulated and blue represents the down-regulated genes. *SbPAO2* and *SbPAO3* genes are upregulated > 2FC under heat and combined stress conditions and *SbPAO1/3/4/6* upregulated under drought. *SbPAO5* is variably expressed at a mild level of downregulation (−2FC) under drought, moderately downregulated (−14FC) under heat stress, and strongly downregulated under combined stress (−26FC). *SbPAO6* downregulated under combined stress conditions (−2.5FC).

A total of 151 genes were identified in co-expression network and 127 of 151 (~ 84%) genes were found to have positive co-expression relationship according to SorghumFDB database (Fig. 7D, Table S8). Many edges in the network prove the broad roles of *SbPAO* genes in cell signaling and metabolism. Interestingly, no miRNAs targeting the *SbPAO* genes were identified in the co-expression network. The first five neighboring genes showing a positive coexpression with the *SbPAO5* genes with the highest Pearson’s correlation coefficient (PCC) score (Table S8). Sobic.001G143000 (NPR1-like protein 3), Sobic.001G450700 (heat shock factor 1), Sobic.009G219900 (Peptidyl-tRNA hydrolase family protein), Sobic.003G358100 (O-fucosyltransferase family protein), and Sobic.006G025300 (ABC transporter family protein) genes were identified as first neighbors for Sobic.006G220200. Similarly, Sobic.001G488800 (Glutaredoxin family protein), Sobic.004G200100 (plant intracellular ras group-related LRR 1), Sobic.008G187200 (NAD(P)-binding Rossmann-fold superfamily protein), Sobic.003G307500 (A20/AN1-like zinc finger family protein), and Sobic.007G151600 (A20/AN1-like zinc finger family protein) were directly connected neighbor genes of *SbPAO6* (Sobic.003G274600). Lastly, Sobic.007G073100 (*SbPAO1*) was found to have PPI with Sobic.002G274800 (non-yellowing 1).

### Sorghum yield and polyamines content in drought-susceptible and tolerant genotypes

**Fig. 8:**
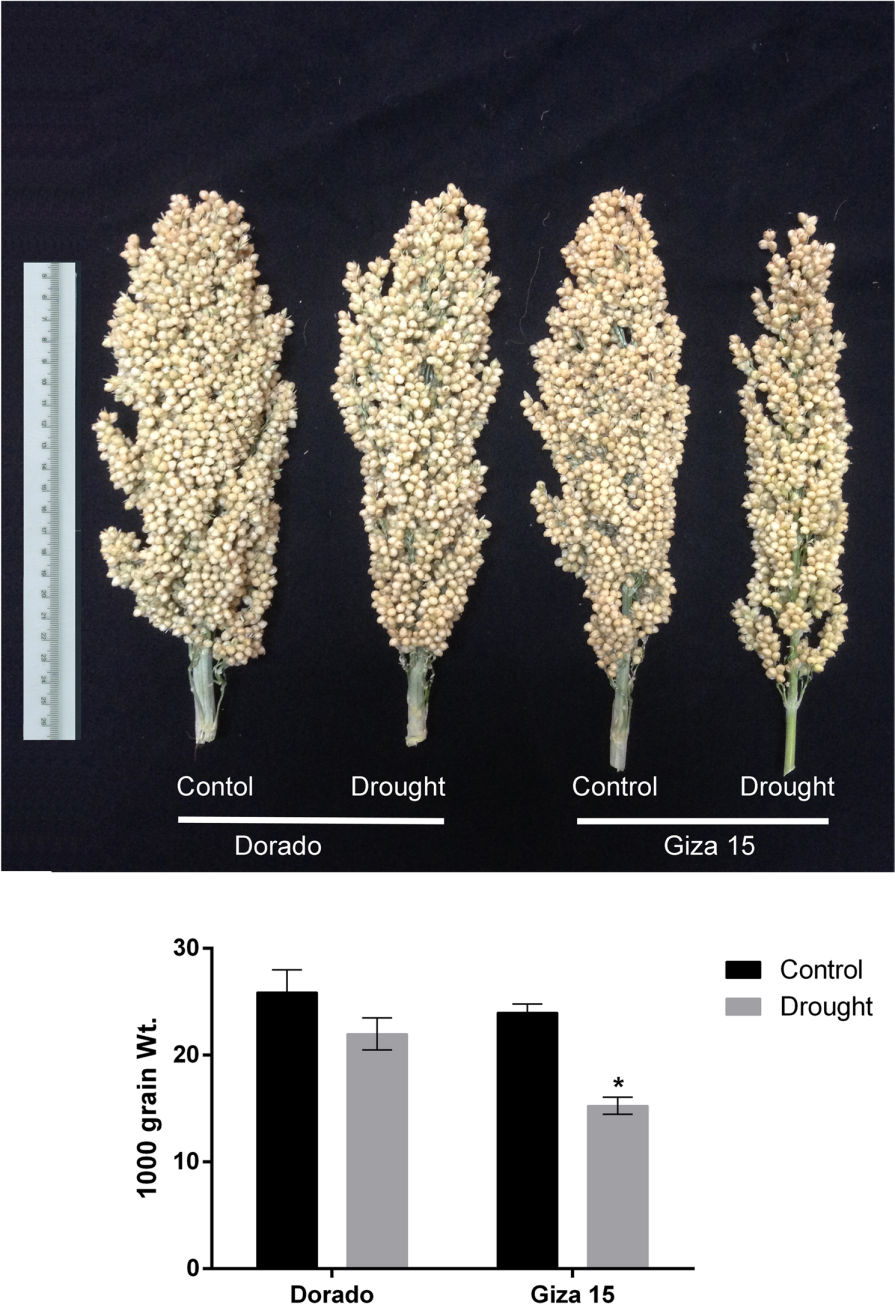
Morphology of heads and weight of 1000 grains (g) in sorghum genotypes Dorado and Giza 15 in control and drought exposed plants. Asterisks indicate significant differences between control and drought treatments within the same genotype (*p < 0.05). Error bars display standard error of the mean (SEM)

**Fig. 9:**
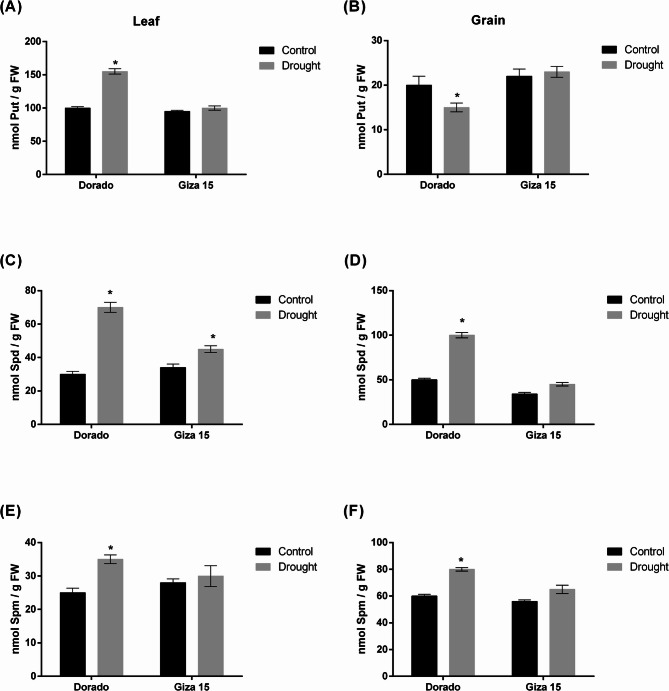
Concentration of free polyamines Putrescine (Put), spermidine (Spd), and spermine (Spm) (nmole/g FW) in leaves and grains of sorghum genotypes Dorado and Giza 15 in control and drought exposed plants. (**A**, **C**, **E**) are Put, Spd, Spm in leaves. (**B**, **D**, **F**) are Put, Spd, Spm in grains. Asterisks indicate significant differences between control and drought treatments within the same genotype (*p < 0.05). Error bars display standard error of the mean (SEM)

Soil moisture was determined at the beginning of withholding water and until the end of the experiment to assure difference between available water in soil for treated plants. Soil water content percentages at the zero time (60-day post sowing) was nearly equal in all plots and decreased after withholding water for drought-exposed plots and reached the minimum at the end of the experiment (Figure S4). 1000 grain weight were declined in both genotypes by drought stress however the more susceptible genotype Giza 15 were sharply declined (Fig. 8). Put content was higher than Spd and Spm in leaves while Spd and Spm were higher than Put in grains was consistent across both genotypes (Fig. 9). The three polyamines’ levels elevated in leaves and grains by drought stress in Dorado genotype but only Spd increased in leaves under drought stress in Giza 15 (Fig. 9).

### Quantitative real-time PCR gene expression analysis for SbPAO genes

**Fig. 10:**
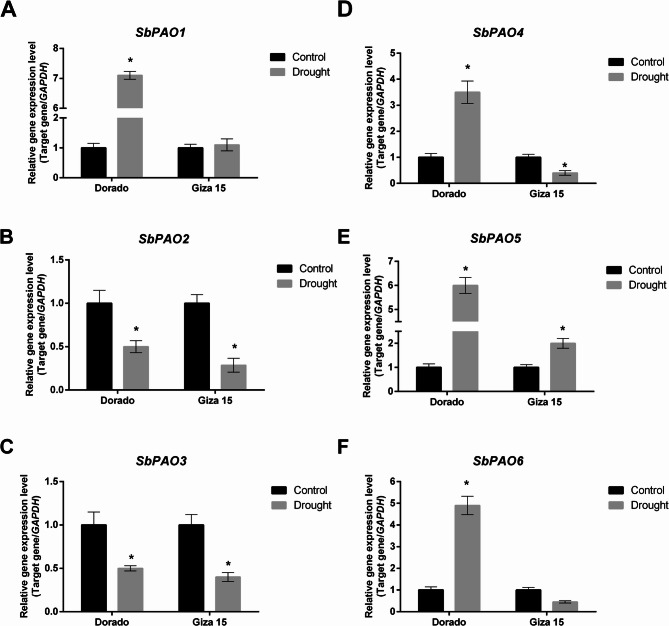
Relatively expressed SbPAO genes in leaves samples of two sorghum genotypes, Dorado and Giza 15, under control and drought conditions. (**A**) Relative expression of SbPAO1 gene in Dorado and Giza 15 leaves, under control and drought conditions. (**B**) Relative expression of SbPAO2 gene in Dorado and Giza 15 leaves, under control and drought conditions. (**C**) Relative expression of SbPAO3 gene in Dorado and Giza 15 leaves, under control and drought conditions. (**D**) Relative expression of SbPAO4 gene in Dorado and Giza 15 leaves, under control and drought conditions. (**E**) Relative expression of SbPAO5 gene in Dorado and Giza 15 leaves, under control and drought conditions. (**F**) Relative expression of SbPAO6 gene in Dorado and Giza 15 leaves, under control and drought conditions. The relative transcript levels were normalized against GAPDH and are shown as fold changes relative to the control. Black bars represent the control, while gray bars represent drought. Asterisks indicate significant differences between control and drought treatments within the same genotype (*p < 0.05). Error bars display standard error of the mean (SEM)

**Fig. 11:**
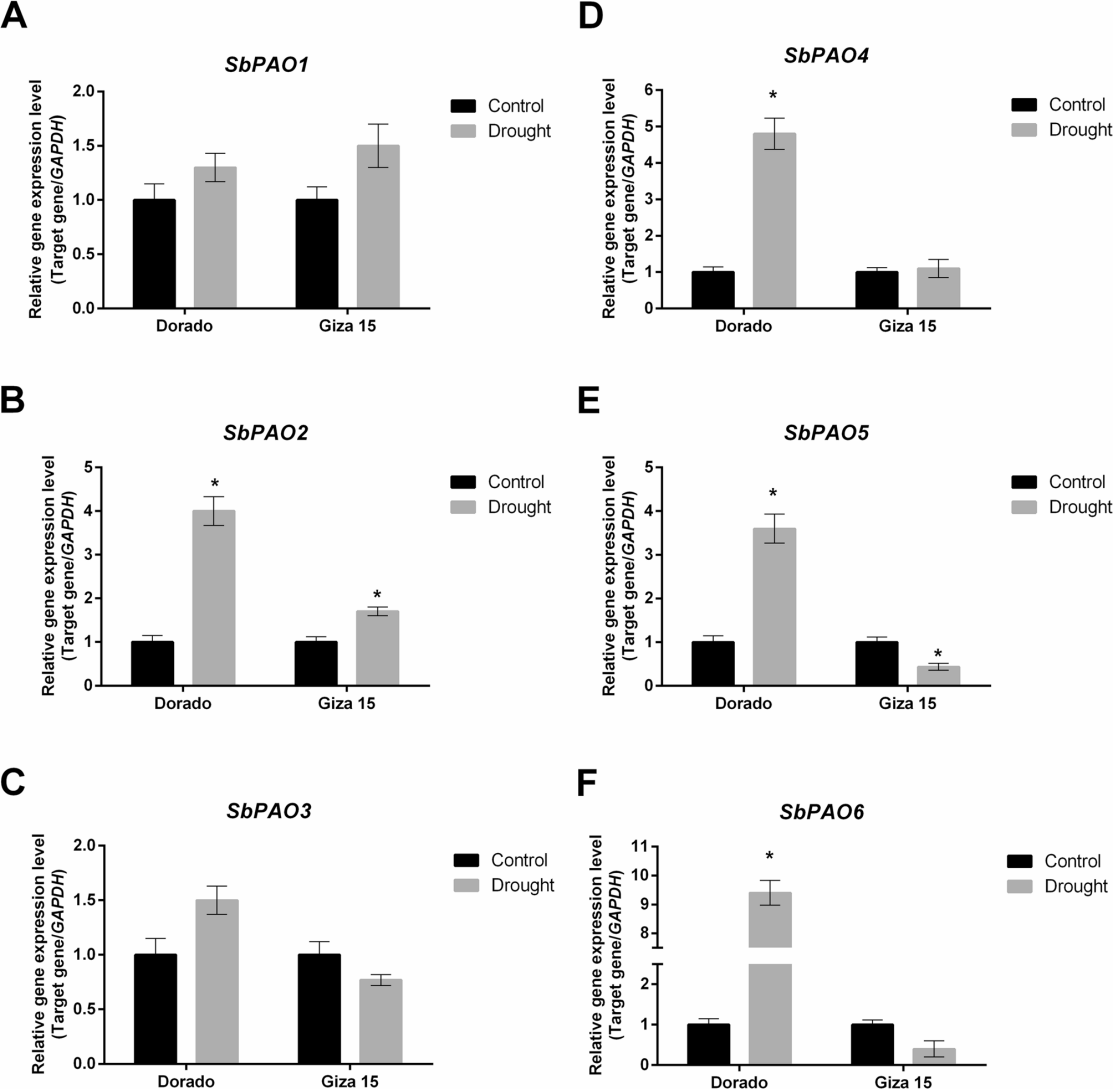
Relatively expressed SbPAO genes in grains of two sorghum genotypes, Dorado and Giza 15, under control and drought conditions. (**A**) Relative expression of SbPAO1 gene in Dorado and Giza 15 grains, under control and drought conditions. (**B**) Relative expression of SbPAO2 gene in Dorado and Giza 15 grains, under control and drought conditions. (**C**) Relative expression of SbPAO3 gene in Dorado and Giza 15 grains, under control and drought conditions. (**D**) Relative expression of SbPAO4 gene in Dorado and Giza 15 grains, under control and drought conditions. (**E**) Relative expression of SbPAO5 gene in Dorado and Giza 15 grains, under control and drought conditions. (**F**) Relative expression of SbPAO6 gene in Dorado and Giza 15 grains, under control and drought conditions. The relative transcript levels were normalized against GAPDH and are shown as fold changes relative to the control. Black bars represent the control, while gray bars represent drought. Asterisks indicate significant differences between control and drought treatments within the same genotype (*p < 0.05). Error bars display standard error of the mean (SEM)

The expression patterns of *SbPAO* genes (*SbPAO1*-*SbPAO6*) were analyzed in leaves and grains of two sorghum genotypes, Dorado and Giza 15, under control and drought conditions (Figs. 10 and 11). In leaves (Fig. 10), the expression of *SbPAO1/4/5* and *SbPAO6* was significantly upregulated in the drought-treated Dorado plants compared to the control. However, in Giza 15, most *SbPAOs* either did not show a significant change between control and drought or downregulated by drought stress. *SbPAO1* and *SbPAO5* showed the highest upregulation in Dorado under drought, with an expression level that was about seven folds higher in comparison to its control. It was also induced under drought conditions in Giza 15, though the induction magnitude was much smaller. While for *SbPAO4*, a significant induction under drought conditions was observed in Dorado, and the expression level was about five folds higher compared to the control. In Giza 15, a significant downregulation (−2.5 FC) in *SbPAO4* expression was detected. Unlike the above-mentioned genes, *SbPAO2* and *SbPAO3* were significantly downregulated upon drought in both the genotypes.

Figure 11 illustrates the relative expressions of *SbPAO* genes, *SbPAO1*-*SbPAO6* in the grains of two sorghum genotypes, namely Dorado and Giza 15, exposed to control and drought stress treatments. The *SbPAO2*, *SbPAO4*, *SbPAO5*, and *SbPAO6* genes under drought stress showed an increased expression profile with high fold changes, especially in *SbPAO6* in the Dorado genotype. On the contrary, these were relatively moderated or unaltered, except the increased expression in *SbPAO2* and the decreased expression of *SbPAO5* in the Giza 15 genotype. Interesting, *SbPAO1* and *SbPAO3* expressions have not changed remarkably in both the genotypes independent of the treatment.

## Discussion

Polyamines are involved in the response to environmental stressors by playing vital roles in numerous plant processes [4, 5, 50]. *PAO* gene family in sorghum has not yet been comprehensively characterized, and the functional roles of its individual members remain unknown [33].

### Structural characteristics reveal functional specialization in SbPAOs

Analysis of PAO proteins in sorghum gave important insights into their structural and functional characteristics. Ten motifs were identified across the six SbPAO proteins, consistent with motif numbers reported in maize [20] and pepper [51]. Four motifs, namely 3, 5, 6, and 7, were conserved in all proteins, three of them corresponding to the Flavin-containing amine oxidoreductase function while the motif no. 6 (GLRLYRTSGDNSVLYDHDLEDYALYDYEGAQVPRETVLK) was found in the six PAO members but not identified in Prosite, Pfam, and InterPro databases which greatly suggests being novel motif for flavine-containing amine oxidoreductase. Although motif analyses in other species noted conserved motif counts and distributions, they did not publish motif sequences; thus, this motif represents a putative novel conserved sequence specific to Sorghum PAOs pending cross-species validation. The MSA of the six SbPAO proteins showed high conservation of the amino acids as glycine, glutamic acid, and proline through multiple sequence alignment in addition to a conserved histidine residue, which is supposed to hydrogen bond with the reactive nitrogen in polyamine substrates, is essential for the efficient oxidation of amines [52]. Predicted 3D structures showed conserved FAD-dependent oxidase cores across all SbPAOs, while SbPAO5 and SbPAO6 exhibited distinct structural elaborations in their folding patterns. These complex folds suggest enhanced protein stability and possible specialized functional roles which is in agree with the results of [53] on soybean, a unique gene structure of *GmPAO3* suggested evolutionary adaptations in polyamine metabolism, crucial for growth, development, and abiotic stress responses.

Furthermore, MSA revealed that protein sequences possess conserved key residues such as lysine, tryptophan, and tyrosine, which are supposed to help in the binding of FAD and stabilization of substrates [27]. PAOs have functional diversity in plants and localized at different subcellular sites. Phylogenetic analysis positions the SbPAOs into four distinct clades with PAO proteins from Arabidopsis, maize, and rice, which indicates evolutionary divergence as well as common ancestry. Proteins of subfamilies I, III, and IV, such as Arabidopsis AtPAO2–AtPAO5, rice OsPAO1, OsPAO3, and OsPAO5 can catalyze the back conversion-reaction of PAs. Subfamily II members such as rice OsPAO2, OsPAO6, OsPAO7, and maize ZmPAO have the ability to catalyze terminal conversion-reaction. Therefore, it could be hypothesized that SbPAO1 and SbPAO2 catalyze the terminal conversion-reaction of PAs in sorghum and the other six proteins could catalyze the back conversion-reaction particularly the SbPAO4 and SbPAO5 which predicted to localize the peroxisome.

Importantly, the structural divergence coincides with subcellular targeting differences: SbPAO4 and SbPAO5 uniquely possess functional peroxisomal targeting signals (PTS1; SRL motif) analogous to AtPAO2-4 [54]. This motif is essential for binding the cytosolic receptor Pex5p, which mediates import into the peroxisomal matrix [16]. The conservation of functional PTS1 signals in specific PAO isoforms suggests evolutionary pressure to compartmentalize polyamine catabolism, likely enabling localized H₂O₂ production for signaling or detoxification while segregating reactive intermediates. The convergence of elaborate folding in SbPAO5 with its peroxisomal localization strongly supports subfunctionalization within this family. Notably, none of the sorghum PAO proteins were predicted to localize to the apoplast, an observation which is in line with the knowledge that PAO enzymes are mainly involved in intracellular polyamine metabolism rather than in extracellular processes [55]. Previous studies on the characterization of the PAO proteins have shown that, in wheat, PAOs are localized in the cytoplasm, while in rice, OsPAO3 to OsPAO5 are reported to be localized in peroxisomes [56], and OsPAO6, OsPAO7 and ZmPAO1 in the apoplast [17, 57]. These results indicate that experimental verification is necessary.

### Evolutionary divergence, extensive gene duplication, and syntenic conservation uncover the genomic organization of PAO genes in Sorghum

PAOs exhibit a broad variability in catalytic properties and subcellular localization, indicating their adaptive evolution across different environments [58]. The evolutionary significance of the *PAO* gene family understood through its phylogenetic relationships, diversity across different species, and its functional roles. The *PAO* gene family in *S. bicolor*, comprising six genes across four chromosomes. Presence of SbPAO proteins in all four clades against retaining only three in other species suggests that sorghum underwent more extensive gene duplication and divergence, retaining all the paralogous copies which may have broader or more diverse physiological capabilities [59]. Chromosomal distribution and synteny analysis pointed out that *SbPAO1* on Chr7 and *SbPAO2* on Chr1 have experienced segmental duplication, which may be indicative of duplication-driven expansion. Tandem duplication was observed for *SbPAO3*, *SbPAO4*, and *SbPAO5* on chromosome 6 is consistent with evolutionary patterns observed in stress-responsive gene families in cereals and Solanaceae [60, 61]. This arrangement may facilitate coordinated expression under environmental challenges, as demonstrated for duplicated *PAO* clusters in rice [15]. Significant gene duplication and loss events contributing to PAO functional diversity [62]. Grass species exhibit large-scale genome collinearity [63]. Interestingly, the analysis of *SbPAO* synteny between sorghum and maize or sorghum and rice revealed multiple relationships suggest that the gene has undergone duplication events in both species. Similarly, gene order conservation on chromosomes 1 and 3 in sorghum and maize indicate collinear functional similarity between species [64]. In the case of sunflowers, chromosomal rearrangements seem to less impact genetic divergence between species, while collinear chromosomes reveal a more striking adaptive divergence [65]. These observations highlight evolutionary constraints preserving the genomic context and support the possible functional redundancy or specialization of *PAO* genes in sorghum.

### Protein–protein interactions, co-expression network, and promoter architecture of SbPAOs

Functional analyses through PPI, promoter elements, and co-expression analysis were performed to uncover important candidates of *SbPAO* genes under drought stress. PPI analysis is important to the understanding of the functional roles of PAO proteins in sorghum. Strong associations among SbPAO proteins and enrichment in polyamine metabolism processes, such as spermine and thermospermine catabolism, suggested their critical role in the homeostasis of polyamines that is, tightly regulating plant growth, development, and stress responses [4–6]. The extended PPI network shows that these interactions are present with proteins participating in wider metabolic processes, such as amine and polyamine metabolism, as well as responding to environmental stimuli. According to Takahashi and Kakehi [66], spermidine is essential for embryo survival and protein synthesis, and spermine plays a role in the stress response through the modulation of cation channels and the production of H_2_O_2_ upon pathogen infection. Thermospermine is an isomer of spermine, which participates in stem elongation [67]. This might imply that PAO proteins in sorghum play a crucial role not only in metabolic pathways but also in modulating plant adaptation to biotic and abiotic stresses.

*SbPAO5* (Sobic.006G220200) shows co-expression with heat shock factor 1 (*HSFA1*), peptidyl-tRNA hydrolase family protein, O-fucosyltransferase family protein, and ABC transporter family protein which indicates a complex system of regulatory integration of different signaling pathways regarding plant stress response. In tea plants, NPR1-like genes take part in hormonal and stress responses, implying that they are involved broadly in stress adaptation [68]. The co-expression of *SbPAO5* with *NPR1-like proteins* and *HSFA1* transcription factors align with established links between polyamine catabolism and stress responses in plants [69]. This network potentially enables H₂O₂-mediated signalling during defence activation, though mechanistic validation is needed. In addition, ABC transporters operate at the cell level to maintain cellular homeostasis through a diverse range of molecule transport across membranes, especially during stress [70]. Likewise, O-fucosyltransferase family proteins that mediate protein glycosylation play a role in stabilizing proteins and maintaining functionality under stress [70]. The co-expression of glutaredoxin (*GRX*) family protein, plant intracellular Ras group-related LRR 1, NAD(P) binding Rossmann-fold superfamily protein, A20/AN1-like zinc finger family protein, and a family of unknown function (DUF716) with *SbPAO6* (Sobic.003G274600) further supports the view that PAOs are involved in stress response and metabolic regulation. Glutaredoxins (*GRXs*) are best known for their roles in redox biology and iron-sulfur cluster assembly, which would be critical for cellular homeostasis maintenance under stress [71, 72]. A20/AN1-like zinc finger and NAD(P)-binding Rossmann-fold proteins are also likely to be involved in stress-responsive signal transduction [73–77].

Analysis of promoter structures and regulatory motifs provide insights into the mechanisms of stress-responsive gene regulation [78]. Presence of key regulatory elements in all *SbPAO* promoters, such as STRE and ARE (stress-responsive), ABRE (hormone-responsive), and Box4 and TCT-motif (light-responsive), suggest that these genes are integral to sorghum ‘s adaptation to environmental changes. Environmental responsive elements were found in all promoters providing evidence for strong role of *SbPAO* in response to stress conditions. STRE, identified to be a striking stress-responsive sequence with the (AAGGGG) core sequence, is implicated in response to various abiotic stimuli [79]. MYB and MYB-binding sites CREs occurred in >80% of *SbPAO* promoters. MYB transcription factors are very important stress response mechanisms influencing expression of genes along different biochemical pathways in plants during stressful encounters [80]. Further, its preponderance within MeJA-responsive elements (such as TGACG-motif and CGTCA-motif) hinted at its possible involvement in the defense and related stress signaling. The presence of two hormone-responsive CREs, the AREB and MeJA responsive elements, point to the role of a hormonal signaling mechanism in the molecular network regulating the modulator functions of PAs in sorghum.

### Polyamine accumulation and grain‑yield under drought in sorghum

A thousand-grain weight has been termed as a good reliable selection trait under irrigation and rainfed situations as attested by its positive correlation with grain yield [81]. A very pronounced drop in a 1000-grain weight as a result of drought further reflects poor grain filling in Giza 15 indicates its susceptibility in contrast to Dorado which is not significantly affected. Studies have indicated the ranges of reduction in 1000-grain weight from 9.2% to 29.9% due to drought condition [81, 82]; nevertheless, drought-resistant hybrids can reduce this effect through better mobilization during grain filling [83]. In drought conditions, all three polyamines significantly increased in leaves and grains of Dorado; only levels of Spd accumulated in leaves of Giza 15. This elevation in Spd levels in Giza leaves could be explained by back conversion-reaction of Spm to Spd by *SbPAO5* which up-regulated in the same condition as indicated from current results. The elevation of polyamines in Dorado probably contributed to its hardiness: Put is an osmoprotectant and substrate for further metabolites, Spd and Spm stabilize macromolecules and scavenge free reactive oxygen species [84]. It is apparent, therefore, that Dorado most likely utilizes the entire array of polyamines at source (leaves) and sink (grains) tissues to counter any potential effects of water stress on cellular homeostasis and the continued filling of grains. On the contrary, such a limited polyamine response would not accede into Giza 15 under drought conditions, thereby suffering larger losses in yield.

### Tissue‑ and genotype‑specific SbPAO expression in response to drought

Gene expression analysis of *SbPAO* genes in sorghum highlighted their possible functional roles in tissue-specific processes and stress responses. Expression profiles indicated differential patterns in different tissues and under stressed conditions, hence showing participation in different biological processes. Tissue-specific expression patterns of *SbPAO3* and *SbPAO4* show the highest levels in reproductive tissues, seed and spikelet. *SbPAO1* is highly expressed only in roots and therefore might participate in root-specific processes related to nutrient uptake and stress perception. In Arabidopsis, *AtPAO2* is responsible for ABA-dependent developmental processes related to root growth and production of nitric oxide; its overexpression increases the growth of the root [85]. *SbPAO3*, *SbPAO4*, and *SbPAO5* are highly expressed in all tissues, which might underpin their multifunctional role in growth and development and enforce their proposed functional redundancy as tandemly duplicated genes, while *SbPAO6* shows a high level of expression in roots and shoots, suggesting its role in vegetative growth. Tissue-specific expression ensures that polyamine levels are tightly regulated in specific tissues [6, 86].

Under stress conditions, the upregulation of *SbPAO2* and *SbPAO3* in transcriptome data by stress from heat, drought, and combined stresses, strongly supports their participation in the amelioration of oxidative damage and polyamine homeostasis under adverse conditions. In wheat, stress-induced activation of the PAO, through heat and drought, results in increased production of H_2_O_2_ and lignification, which might contribute to stress tolerance [87]. Interestingly, enhanced Arabidopsis tolerance to salt and drought imposed by the elimination of cytoplasmic PAO activity was described, with its possible causes being decreased ROS production and upregulation of stress-related genes [88]. This might indicate certain differences in the regulation mechanisms and functional specialization of the *SbPAO* genes under abiotic stress.

Genotype-dependant response to drought revealed differential *SbPAO* gene expression patterns of responses specific to both the genotype and organs in leaf and grain organs within two genotypes Dorado and Giza 15. Results of leaves tissues indicated a strong drought-induced response in Dorado with dynamic transcription that responds greatly under significant inductions of several *PAO* genes (*SbPAO4*, *SbPAO5*, and *SbPAO6*) suggesting their critical roles in enhancing stress resilience. Contrarily, downregulation of *SbPAO2/4* and *SbPAO6* in Giza 15 implies a weaker or less consistent expression response in leaves and grains, which might be linked to its lesser drought tolerance. The consistent downregulation of *SbPAO3* in both genotypes although up-regulation in response to stress in transcriptome data are in agree with Li et al. [89] who revealed distinct expression patterns of seven *Camellia sinensis PAO* under abiotic stress conditions and could be explained by Samanta et al. [90] suggestion that in some species, *PAO* genes are expressed differentially with spatial and temporal distinctions, further highlighting their tissue-specific roles in stress responses. In grains, the marked induction of *SbPAO6* in Dorado suggests a pivotal function in maintaining grain viability and metabolic homeostasis under drought conditions. The upregulation of *SbPAO6* under stress align with the complex 3D structure of SbPAO6 protein.

These results therefore indicate that *SbPAO5* and *SbPAO6* genes, are promising candidates for improvement against drought. Based on their strong upregulation under drought stress, co-expression with stress-responsive genes, and their unique structural features. These findings offer potential targets for genetic improvement of drought tolerance through marker-assisted breeding or biotechnological approaches.

## Conclusions

This study provides new insights into the evolutionary trajectory, structural divergence, and functional specialization of the polyamine oxidase gene family that are involved in the degradation of polyamine in sorghum. The study six SbPAO genes with diverse subcellular localizations and structural differences, suggesting possible variation in substrate specificity and catalytic activity. Phylogenetic and synteny analyses revealed both segmental (SbPAO1/2) and tandem (SbPAO3/4/5) duplications, indicating gene family expansion and conservation across sorghum, maize, and rice. Promoter analysis highlighted the presence of hormone- and stress-responsive cis-elements, suggesting transcriptional regulation of SbPAOs under abiotic stress. Expression profiling demonstrated tissue- and genotype-specific regulation, with drought-tolerant Dorado showing upregulation of SbPAO4–6, polyamine accumulation, and stable yield, in contrast to the sensitive genotype Giza 15. These findings not only advance our understanding of *PAO* gene evolution in grasses but also provide actionable targets for molecular breeding. Future efforts to validate *SbPAO5* and *SbPAO6* through CRISPR-based editing or overexpression could unlock their potential in enhancing crop productivity under climate-driven abiotic stresses.

## Supplementary Information

Supplementary Material 1.

Supplementary Material 2.

Supplementary Material 3.

Supplementary Material 4.

Supplementary Material 5.

Supplementary Material 6.

Supplementary Material 7.

Supplementary Material 8.

Supplementary Material 9.

Supplementary Material 10.

Supplementary Material 11.

Supplementary Material 12.

## Data Availability

Data is provided within the manuscript or supplementary files.

## Abbreviations

PAOs: Polyamine oxidases
SbPAOs: Sorghum bicolor polyamine oxidases
Put: Putrescine
Spd: Spermidine
Spm: Spermine
SRA: Sequence Read Archive
TPM: Transcript Per Million
FC: Fold change
LSD: Least significant difference
CREs: Cis-acting regulatory elements
