# Supplementary material for: Comparative genomics and expression analysis of polyamine oxidase gene family in Sorghum bicolor reveals functional specialization, gene duplication, and role in drought resilience
Source: BMC Genomics. 2025 Oct 28;26:966. doi: 10.1186/s12864-025-12125-4 (PMC12570722; doi:10.1186/s12864-025-12125-4)
Supplement: Supplementary file 1 — Supplementary Material 1. [file 12864_2025_12125_MOESM1_ESM.docx]

**Table S1: Primer sequences used in the study for Sorghum PAO genes.**

| **Name** | **Primer pairs** | **Sequence (5'->3')** | **Length** | **Tm** | **GC%** | **Product size** |
| --- | --- | --- | --- | --- | --- | --- |
| ***SbPAO1***  Sobic.007G073100 | Fw | 5'- CAACAAGGTGGTGCGAGAAA -3' | 20 | 58.98 | 50 | 76 |
|  | Rv | 5'- CCTGTACACCGAGTTGTCCT-3' | 20 | 59.03 | 55 |  |
| ***SbPAO2***  Sobic.001G472000 | Fw | 5'- ACGGTTATGTCCATGGAGCA-3' | 20 | 59.09 | 59.09 | 91 |
|  | Rv | 5'- CGGACCTTGTACTCCTCGTT-3' | 20 | 59.11 | 59.11 |  |
| ***SbPAO3***  Sobic.006G261500 | Fw | 5'- AGCGTGTTCTTACTGGTGGA-3' | 20 | 58.95 | 50 | 117 |
|  | Rv | 5'- GGCGGGTAATTTCGGTAACC-3' | 20 | 58.98 | 55 |  |
| ***SbPAO4***  Sobic.006G261600 | Fw | 5'- AAGTGCTAGGTAGGATCGCC-3' | 20 | 58.96 | 55 | 110 |
|  | Rv | 5'- TGCGAATCTTCCAGCTACCA-3' | 20 | 59.1 | 50 |  |
| ***SbPAO5***  Sobic.006G220200 | Fw | 5'- GATCCTTCCCAATGCAGCTG-3' | 58.97 | 58.97 | 55 | 96 |
|  | Rv | 5'- TTCACCCCATCGAACGTGTA-3' | 59.03 | 59.03 | 50 |  |
| ***SbPAO6***  Sobic.003G274600 | Fw | 5'- AGGGTGAAGCGGATCAAGAG-3' | 20 | 59.46 | 55 | 70 |
|  | Rv | 5'- CCACGTAGCTGTAGGACCC-3' | 19 | 59.19 | 63.16 |  |
| Ref gene  **GAPDH** | Fw | 5'-TTGCTCTGAACGACCATTTC-3' | 20 | 60.7 | 45 | 175 |
|  | Rv | 5'-GACACCATCCACATTTATTCTTC-3' | 23 | 59.8 | 39.1 |  |
