# Supplementary material for: Comparative genomics and expression analysis of polyamine oxidase gene family in Sorghum bicolor reveals functional specialization, gene duplication, and role in drought resilience
Source: BMC Genomics. 2025 Oct 28;26:966. doi: 10.1186/s12864-025-12125-4 (PMC12570722; doi:10.1186/s12864-025-12125-4)
Supplement: Supplementary file 2 — Supplementary Material 2. [file 12864_2025_12125_MOESM2_ESM.docx]

**Table S2.** Description of the conserved motifs identified by MEME in the PAO proteins.

| **Motif No.** | **Motif sequence** | **Width** | **Description** |
| --- | --- | --- | --- |
| 1 | PLGVLKANLIKFEPZLPAWKISAIYDLGVGVENKIALKFPKVFWPNVE | 48 | Flavin containing amine oxidoreductase |
| 2 | RWGSDPNSLGSYSNDPVGKPRDLYERLRAPVGNLYFAGEATSEKYSGSVH | 50 | Flavin containing amine oxidoreductase |
| 3 | DVTILEARDRIGGRVHTDYFFGIPVDMGASWLHGVCGEN | 39 | Flavin containing amine oxidoreductase |
| 4 | LQWCICRLEAWFATDADNISLKNWDQERVLTGGHGLMVNGYDPVIEALAQ | 50 | NI |
| 5 | TGNPVLLVMVAGREAREIEKLSDEEAVAFVMSQLRKMLP | 39 | Flavin containing amine oxidoreductase |
| 6 | GLRLYRTSGDNSVLYDHDLEDYALYDYEGAQVPRETVLK | 39 | NI |
| 7 | PRVIVIGGGISGIAAAKALSNAGF | 24 | Flavin containing amine oxidoreductase |
| 8 | LNKRVTEIVRHYNGVVVTTEDGSSYVAD | 28 | Flavin containing amine oxidoreductase |
| 9 | FEKILEETVKVRDEQENDMPLJQAIAIVLDRNPHLKLEG | 39 | NI |
| 10 | GAYSSGIAAAEECRNRLLKK | 20 | NI |

**^*NI: Not identified by motifs databases.^**
