# Supplementary material for: Comparative genomics and expression analysis of polyamine oxidase gene family in Sorghum bicolor reveals functional specialization, gene duplication, and role in drought resilience
Source: BMC Genomics. 2025 Oct 28;26:966. doi: 10.1186/s12864-025-12125-4 (PMC12570722; doi:10.1186/s12864-025-12125-4)
Supplement: Supplementary file 5 — Supplementary Material 5. [file 12864_2025_12125_MOESM5_ESM.docx]

| **Species** | **Name** | Gene ID |
| --- | --- | --- |
| *Zea mays* | *ZmPAO1* | Zm00001d024281_T001 |
|  | *ZmPAO2* | Zm00001d043681_T001 |
|  | *ZmPAO3* | Zm00001d001883_T001 |
|  | *ZmPAO4* | Zm00001d026586_T003 |
|  | *ZmPAO5* | Zm00001d028172_T001 |
|  | *ZmPAO6* | Zm00001d026334_T001 |
|  | *ZmPAO7* | Zm00001d037642_T001 |
|  | *ZmPAO8* | Zm00001d036513_T001 |
|  | *ZmPAO9* | Zm00001d002266_T001 |
| *Arabidopsis thaliana* | *AtPAO1* | AT5G13700.1 |
|  | *AtPAO2* | AT2G43020.1 |
|  | *AtPAO3* | AT3G59050.1 |
|  | *AtPAO4* | AT1G65840.1 |
|  | *AtPAO5* | AT4G29720.1 |
| *Sorghum bicolor* | *SbPAO1* | Sobic.007G073100 |
|  | *SbPAO2* | Sobic.001G472000 |
|  | *SbPAO3* | Sobic.006G261500 |
|  | *SbPAO4* | Sobic.006G261600 |
|  | *SbPAO5* | Sobic.006G220200 |
|  | *SbPAO6* | Sobic.003G274600 |
| *Oryza sativa* | *OsPAO1* | LOC_Os01g51320 |
|  | *OsPAO2* | LOC_Os03g09810 |
|  | *OsPAO3* | LOC_Os04g53190 |
|  | *OsPAO4* | LOC_Os04g57550 |
|  | *OsPAO5* | LOC_Os04g57560 |
|  | *OsPAO6* | LOC_Os09g20260 |
|  | *OsPAO7* | LOC_Os09g20284 |
| *Solanum lycopersicum* | *SlPAO1* | Solyc01g087590 |
|  | *SlPAO2* | Solyc07g043590 |
|  | *SlPAO3* | Solyc12g006370 |
|  | *SlPAO4* | Solyc02g081390 |
|  | *SlPAO5* | Solyc03g031880 |
|  | *SlPAO6* | Solyc07g039310 |
|  | *SlPAO7* | Solyc05g018880 |
| *Citrus sinensis* | *CsPAO1* | Cs7g02060.1 |
|  | *CsPAO2* | Cs7g18840.2 |
|  | *CsPAO3* | Cs6g15870.1 |
|  | *CsPAO4* | Cs4g14150.1 |
|  | *CsPAO5* | Cs7g23790.1 |
|  | *CsPAO6* | Cs7g23670.1 |
| *Linum usitatissimum* | *LuPAO1* | Lus10020726 |
|  | *LuPAO2* | Lus10005021 |
|  | *LuPAO3* | Lus10039599 |
|  | *LuPAO4* | Lus10029495 |
|  | *LuPAO5* | Lus10019725 |
| *Solanum tuberosum L.* | *StPAO1* | Soltu.DM.01G027080 |
|  | *StPAO2* | Soltu.DM.03G002990 |
|  | *StPAO3* | Soltu.DM.12G023960 |
|  | *StPAO4* | Soltu.DM.07G014310 |
|  | *StPAO5* | Soltu.DM.02G020890 |
|  | *StPAO6* | Soltu.DM.10G012870 |
|  | *StPAO7* | Soltu.DM.07G024810 |
|  | *StPAO8* | Soltu.DM.04G036220 |
|  | *StPAO9* | Soltu.DM.05G026290 |
|  | *StPAO10* | Soltu.DM.11G004390 |
|  | *StPAO11* | Soltu.DM.05G013880 |
|  | *StPAO12* | Soltu.DM.07G011640 |
|  | *StPAO13* | Soltu.DM.03G016020 |
|  | *StPAO14* | Soltu.DM.12G025820 |
| *Cucumis sativus* L | *CsaPAO1* | CsaV3_7G030830 |
|  | *CsaPAO2* | CsaV3_4G007490 |
|  | *CsaPAO3* | CsaV3_2G001470 |
|  | *CsaPAO4* | CsaV3_4G032140 |
| *Saccharomyces cerevisiae* | *FMS1* | SGD:S000004622 |
