# Supplementary material for: Comparative genomics and expression analysis of polyamine oxidase gene family in Sorghum bicolor reveals functional specialization, gene duplication, and role in drought resilience
Source: BMC Genomics. 2025 Oct 28;26:966. doi: 10.1186/s12864-025-12125-4 (PMC12570722; doi:10.1186/s12864-025-12125-4)
Supplement: Supplementary file 7 — Supplementary Material 7. [file 12864_2025_12125_MOESM7_ESM.docx]

**Table S6**. The total sum of cis elements within each category of CREs in *SbPAO* promoters.

|  | ***SbPAO1*** | ***SbPAO2*** | ***SbPAO3*** | ***SbPAO4*** | ***SbPAO5*** | ***SbPAO6*** |
| --- | --- | --- | --- | --- | --- | --- |
| Sum of LRE | 10 | 9 | 9 | 9 | 15 | 9 |
| Sum of HRE | 15 | 9 | 8 | 9 | 12 | 13 |
| Sum of ERE | 17 | 9 | 9 | 9 | 11 | 12 |
| Sum of DRE | 7 | 5 | 9 | 5 | 12 | 9 |
| Sum of PE | 117 | 190 | 154 | 152 | 74 | 59 |
| Sum of SBE | 7 | 3 | 5 | 3 | 6 | 10 |
| Total sum of elements | 189 | 236 | 118 | 197 | 157 | 124 |

^LRE: Light-responsive elements; HRE: Hormone-responsive elements; ERE: Environment-responsive elements; DRE: Development-responsive elements; PE: Promoter elements; SBE: Site binding elements.^
