# Supplementary material for: Comparative genomics and expression analysis of polyamine oxidase gene family in Sorghum bicolor reveals functional specialization, gene duplication, and role in drought resilience
Source: BMC Genomics. 2025 Oct 28;26:966. doi: 10.1186/s12864-025-12125-4 (PMC12570722; doi:10.1186/s12864-025-12125-4)
Supplement: Supplementary file 8 — Supplementary Material 8. [file 12864_2025_12125_MOESM8_ESM.docx]

**Table S5**. Numbers of elements within each category of CREs in *SbPAO* promoters.

|  | ***SbPAO1*** | ***SbPAO2*** | ***SbPAO3*** | ***SbPAO4*** | ***SbPAO5*** | ***SbPAO6*** |
| --- | --- | --- | --- | --- | --- | --- |
| Count of LRE | 6 | 5 | 7 | 5 | 7 | 6 |
| Count of HRE | 7 | 7 | 4 | 7 | 6 | 8 |
| Count of ERE | 9 | 7 | 6 | 7 | 6 | 7 |
| Count of DRE | 3 | 3 | 4 | 3 | 7 | 6 |
| Count of PE | 7 | 8 | 8 | 6 | 9 | 8 |
| Count of SBE | 5 | 2 | 2 | 2 | 3 | 4 |
| Total count of elements | 38 | 34 | 34 | 31 | 41 | 41 |

^LRE: Light-responsive elements; HRE: Hormone-responsive elements; ERE: Environment-responsive elements; DRE: Development-responsive elements; PE: Promoter elements; SBE: Site binding elements.^
