# Supplementary figures and images for: Comparative genomics and expression analysis of polyamine oxidase gene family in Sorghum bicolor reveals functional specialization, gene duplication, and role in drought resilience
Source: BMC Genomics. 2025 Oct 28;26:966. doi: 10.1186/s12864-025-12125-4 (PMC12570722; doi:10.1186/s12864-025-12125-4)

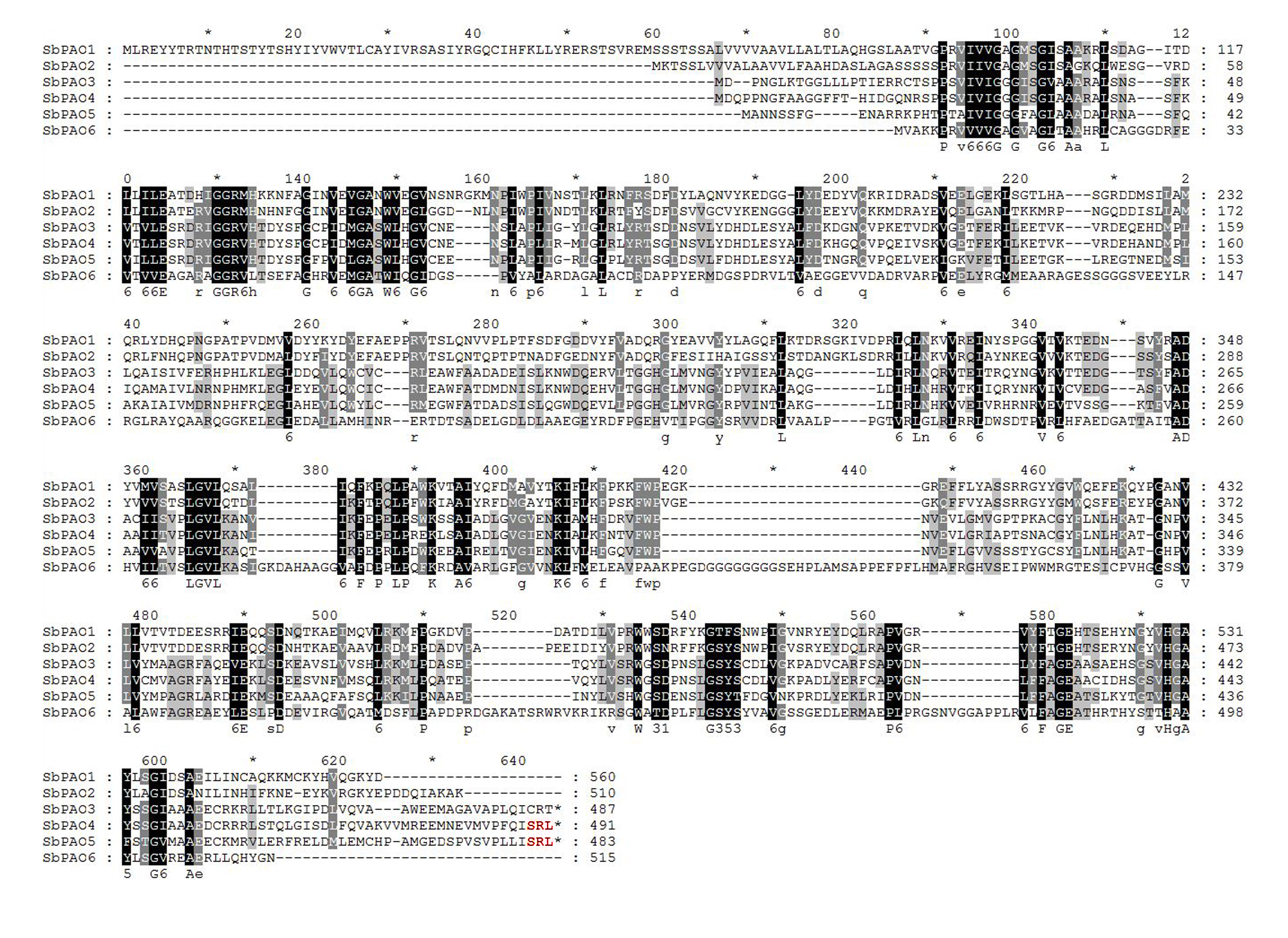

Supplement: Supplementary file 10 — Supplementary Material 10. [file 12864_2025_12125_MOESM10_ESM.jpg]

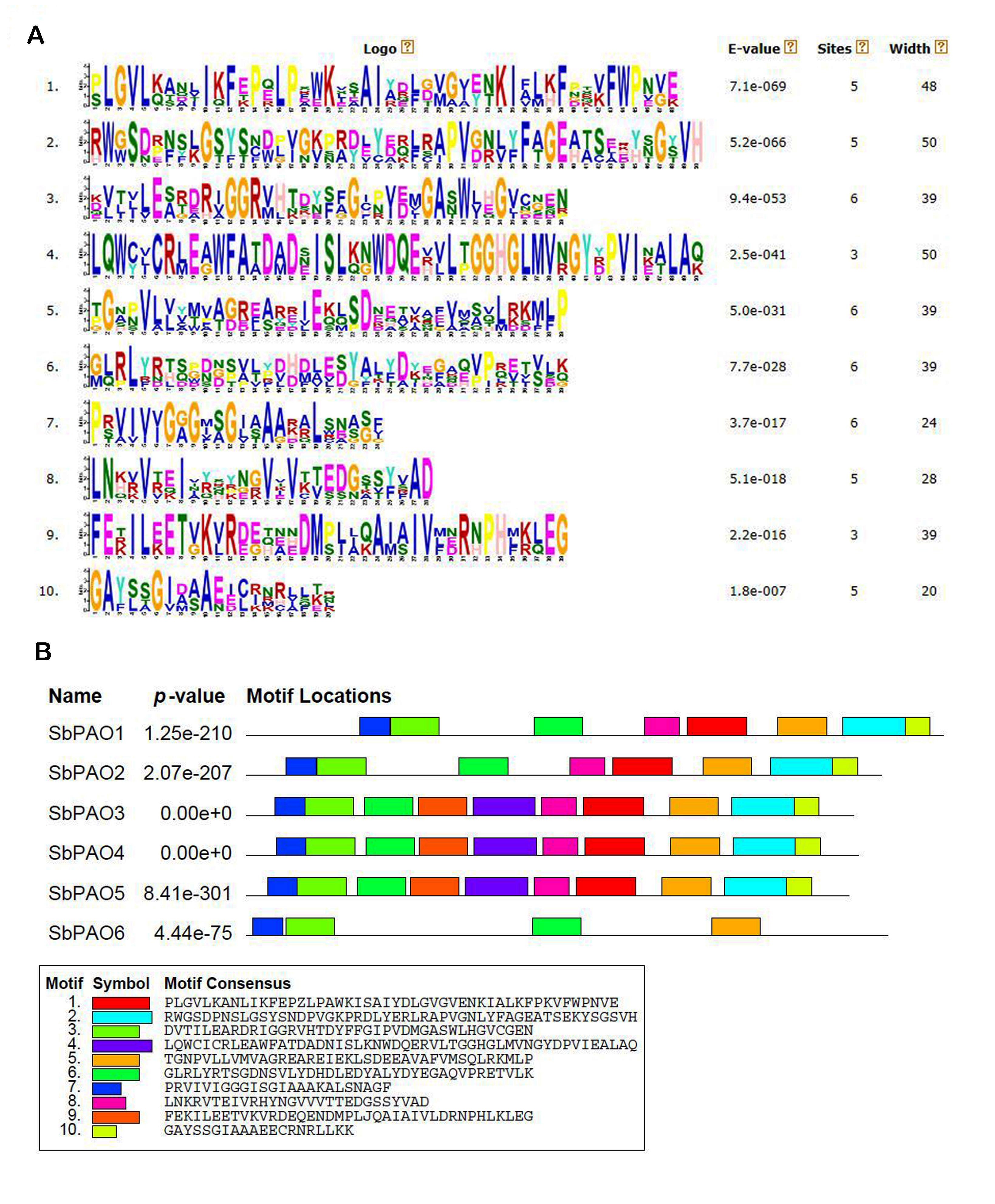

Supplement: Supplementary file 11 — Supplementary Material 11. [file 12864_2025_12125_MOESM11_ESM.jpg]

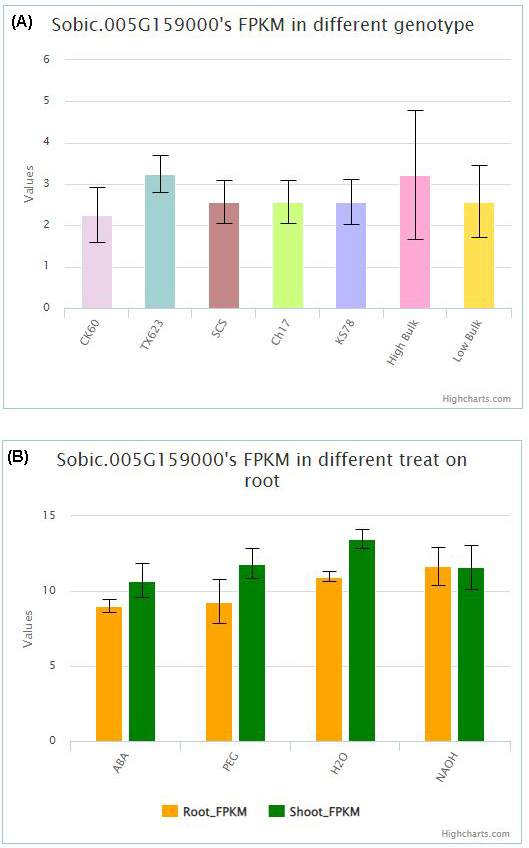

Supplement: Supplementary file 12 — Supplementary Material 12. [file 12864_2025_12125_MOESM12_ESM.jpg]
